# First-principles investigation of optoelectronic, thermoelectric, and photocatalytic properties of Ca_3_Zr_1−*x*_Sn_*x*_Si_2_O_9_

**DOI:** 10.1039/d6ra02396g

**Published:** 2026-07-02

**Authors:** Oumnia Racha Selmi, Rachid Makhloufi, Rania Charif, Ali Ismael, Taha Abdel Mohaymen Taha

**Affiliations:** a Laboratory of Applied Chemistry (LCA), University of Biskra PO Box 145 07000 Biskra Algeria oumniaracha.selmi@univ-biskra.dz r.makhloufi@univ-biskra.dz; b Physics Department, Lancaster University Lancaster LA1 4YB UK k.ismael@lancaster.ac.uk; c Physics and Engineering Mathematics Department, Faculty of Electronic Engineering, Menoufia University Menouf 32952 Egypt taha.hemida@yahoo.com

## Abstract

A combined experimental and first-principles investigation was carried out to study the structural, vibrational, microstructural, electronic, optical, photocatalytic, and thermoelectric properties of Ca_3_Zr_1−*x*_Sn_*x*_Si_2_O_9_ (*x* = 0, 0.5, 1). X-ray diffraction confirms the preservation of the monoclinic *P*2_1_/*c* baghdadite-type framework across the series. Raman spectroscopy reveals a systematic red-shift of the characteristic Zr–O–Zr bending mode from 620 cm^−1^ (*x* = 0) to 603 cm^−1^ (*x* = 1), with coexistence of both modes at *x* = 0.5, indicating mixed local environments. SEM observations revealed that all samples maintain a similar irregular plate-like morphology, while EDS analysis confirms compositional homogeneity. Complementary DFT calculations were performed using GGA-PBE and HSE06 to refine the electronic properties. Structural optimization and elastic constants confirm mechanical stability for all compositions. Sn substitution significantly modifies the conduction-band character and systematically reduces the electronic band gap: the HSE06-calculated gaps decrease from 5.076 eV (*x* = 0) to 4.047 eV (*x* = 0.5) and 3.903 eV (*x* = 1). This trend agrees with the DRS measurements, which likewise show a measurable reduction of the indirect optical band gap upon Sn incorporation. Strong UV absorption is revealed by the optical calculations, suggesting potential applications in optoelectronic devices. Band-edge positions relative to the normal hydrogen electrode confirmed that both conduction and valence bands straddle the water redox potentials, indicating the feasibility of photocatalytic water splitting under UV excitation. Additionally, the thermoelectric analysis shows encouraging power factors and Seebeck coefficients, underscoring the materials' potential for energy conversion applications. These results highlight Sn substitution as an effective route to tune the electronic structure and functional behavior of Ca_3_ZrSi_3_O_9_, offering potential for energy-related applications including UV-driven photocatalysis and high-temperature thermoelectric conversion.

## Abstract 2

A combined experimental and first-principles investigation was carried out to study the structural, vibrational, microstructural, electronic, optical, photocatalytic, and thermoelectric properties of Ca_3_Zr_1−*x*_Sn_*x*_Si_2_O_9_ (*x* = 0, 0.5, 1).

## Graphical abstract

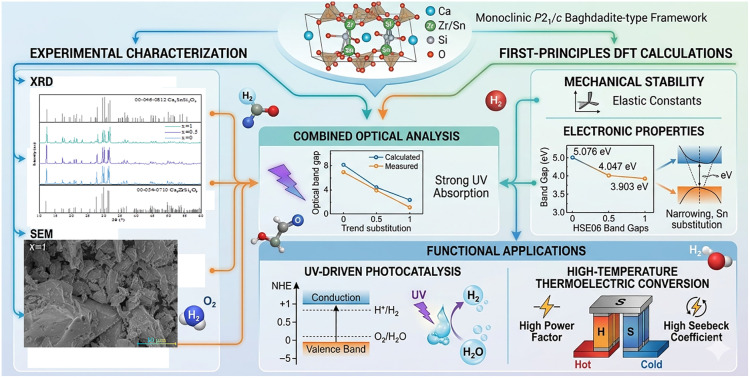

## 1. Introduction

Calcium silicates are foundational materials in ceramic science due to their abundance, intrinsic thermal stability, and wide range of applications, including bioceramics, construction materials, photonic, and dielectric devices.^1–3^ Composed primarily of calcium and silicate units, they exhibit mechanical strength, chemical durability, and structural adaptability, making them ideal host lattices for functional modifications and advanced material design.^4,5^ Beyond their base forms, calcium silicate-based ceramics demonstrate enhanced functional versatility when modified through the incorporation of metal cations.^6,7^ In particular, the substitution of calcium by divalent or tetravalent metal ions leads to the formation of calcium disilicates with tailored physical and chemical properties.^8–10^ Notable examples include CaMgSi_2_O_6_ (diopside), Ca_2_ZnSi_2_O_7_ (hardystonite), and Ca_3_ZrSi_2_O_9_ (baghdadite), each exhibiting application-specific advantages depending on the nature of the substituting cation and the resulting crystal structure.^11^ Among calcium disilicates, Ca_3_ZrSi_2_O_9_ (baghdadite) has attracted considerable interest due to its structural stability and compositional flexibility, making it a promising candidate for advanced ceramic applications.^12–16^ It has been synthesized using various methods, including sol–gel techniques,^17^ and solid-state reactions,^18^ typically requiring high-temperature treatment. Its structural analog Ca_3_SnSi_2_O_9_, although less studied, shares a comparable crystal structure and has been successfully synthesized *via* solid-state methods.^19^ Its potential for dielectric and optical applications arises from the unique electronic configuration and polarizability of Sn^4+^, which can influence the material's bonding and dielectric behavior.^20^ The structural compatibility between these two end-member compounds, combined with their distinct functional characteristics, suggests that forming a Ca_3_Zr_1−*x*_Sn_*x*_Si_2_O_9_ solid solution a promising system for compositional and property tuning.

To date, only a single experimental study has reported the synthesis and crystal structure of the Ca_3_Zr_1−*x*_Sn_*x*_Si_2_O_9_ solid solution,^21^ focusing primarily on its microwave dielectric behavior and structural evolution with varying Sn content. However, a detailed theoretical understanding of how Sn substitution alters the fundamental properties of this system remains lacking, particularly at the atomic scale.

In this work, we combine first-principles density functional theory (DFT) calculations with experimental characterization (XRD, Raman spectroscopy, SEM/EDS, and UV-vis diffuse reflectance) to comprehensively investigate the Ca_3_Zr_1−*x*_Sn_*x*_Si_2_O_9_ (*x* = 0, 0.5, 1.0) solid solution. Theoretical calculations elucidate the influence of Sn incorporation on the structural stability, band gap evolution, dielectric response, and thermoelectric transport properties. Experimentally, solid-state synthesis and optical measurements validate the predicted compositional trends and band gap behavior. Furthermore, band-edge alignment analysis demonstrates the suitability of these oxides for UV-driven photocatalytic water splitting, linking their tunable electronic structure to potential applications in energy conversion and multifunctional oxide technologies.

## 2. Methodology

### 2.1. Sample preparation

The Ca_3_Zr_1−*x*_Sn_*x*_Si_2_O_9_ (*x* = 0, 0.50, and 1) compounds were synthesized *via* the conventional solid-state reaction method. The compositions (*x* values) correspond to the nominal stoichiometric ratios used during synthesis. High-purity analytical-grade precursors: CaCO_3_, ZrO_2_, SnO_2_, and SiO_2_ (Biochem, ≥99%), were weighed according to stoichiometric ratios and thoroughly mixed using a porcelain mortar and pestle with acetone as a dispersing medium. The powders were first precalcined at 1100 °C for 12 h, with heating rate 5 °C min^−1^, in ambient air to decompose carbonates and initiate solid-state reactions, enhancing homogeneity and phase development. After regrinding, they were calcined again at 1430 °C for 24 h (5 °C min^−1^) to achieve full phase formation and crystallization. The furnace was allowed to cool naturally to ambient temperature.

### 2.2. Characterization techniques

The crystal structure of the prepared samples was characterized by X-ray diffraction (XRD) using a Rigaku MiniFlex diffractometer equipped with Cu Kα radiation (*λ*_Kα1_ = 1.5406 Å, *λ*_Kα2_ = 1.5444 Å). The diffraction patterns were recorded over the 2*θ* range of 10–60° with a step size 0.01°, suitable for accurate phase identification and pattern refinement.

The Raman spectra were recorded using a *Renishaw inVia* Reflex micro-Raman spectrometer equipped with a 633 nm laser source. Raman measurements were performed at room temperature to investigate the local structural order, vibrational modes, and possible distortions induced by Sn substitution.

The surface morphology and elemental composition of the samples were examined using scanning electron microscopy (SEM) coupled with energy-dispersive X-ray spectroscopy (EDS) (Hitachi TM3030Plus).

The optical properties were investigated by diffuse reflectance spectroscopy (DRS) using a Shimadzu UV-2600i UVVis-NIR spectrometer operating in the wavelength range of 200–1400 nm. The reflectance data were subsequently transformed using the Kubelka–Munk function to estimate the optical band gap energy.

### 2.3. Computational details

**Fig. 1:**
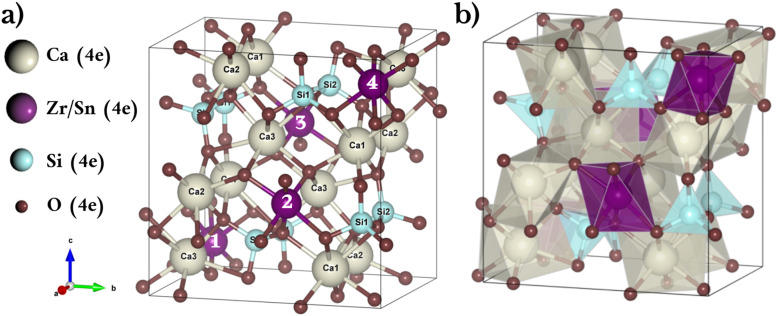
Monoclinic Ca_3_(Zr/Sn)Si_2_O_9_ unit cell structure (a) ball and stick representation, (b) polyhedral representation.

First-principles calculations based on density functional theory (DFT) were performed using the *Quantum ESPRESSO* package.^22^ The exchange–correlation energy was treated within the generalized gradient approximation (GGA) using the Perdew–Burke–Ernzerhof (PBE) functional,^23^ and electron–ion interactions were treated with projector augmented-wave (PAW) pseudopotentials.^24^ In order to calculate the energy band more accurately, the band gap values were calculated using HSE06 (ref. 25) to confirm the results being reliable. A plane-wave energy cutoff of 46 Ry and a charge density cutoff of 380 Ry were adopted. Brillouin zone integrations employed a Monkhorst–Pack^26^*k*-point mesh of 4 × 3 × 3. For each composition (*x* = 0.0, 0.5, 1.0) in Ca_3_Zr_1−*x*_Sn_*x*_Si_2_O_9_, full geometry optimization was carried out by simultaneously relaxing the lattice parameters and atomic coordinates until the total energy, forces, and stresses satisfied the convergence criteria. The optimized structures were subsequently used to determine the equilibrium lattice parameters, unit-cell volumes, and densities. Electronic properties were evaluated from calculated band structures and total/projected density of states (DOS) along high-symmetry paths. Optical properties were obtained from the frequency-dependent dielectric function using the epsilon.x module, while elastic constants were computed *via* the finite distortion approach implemented in the thermo_pw.x code. Thermoelectric coefficients were computed using BoltzTraP2 (ref. 27) based on interpolated band structures. The monoclinic unit cell of Ca_3_ZrSi_2_O_9_ and Ca_3_SnSi_2_O_9_ within the 60-atom structure, is shown in Fig. 1(a). Every atom in the structure is situated on a 4e Wyckoff site, which is a general position with no special symmetry. Corner-sharing [SiO_4_] tetrahedra connect with [ZrO_6_]/[SnO_6_] octahedra and [CaO_6_]/[CaO_7_] polyhedra, forming a three-dimensional framework comprising three distinct Ca^2+^ sites, two Si^4+^ sites and one unique Zr^4+^/Sn^4+^ octahedral site. The polyhedral network is depicted in Fig. 1(b).

## 3. Results and discussion

### 3.1. Structural properties

**Fig. 2:**
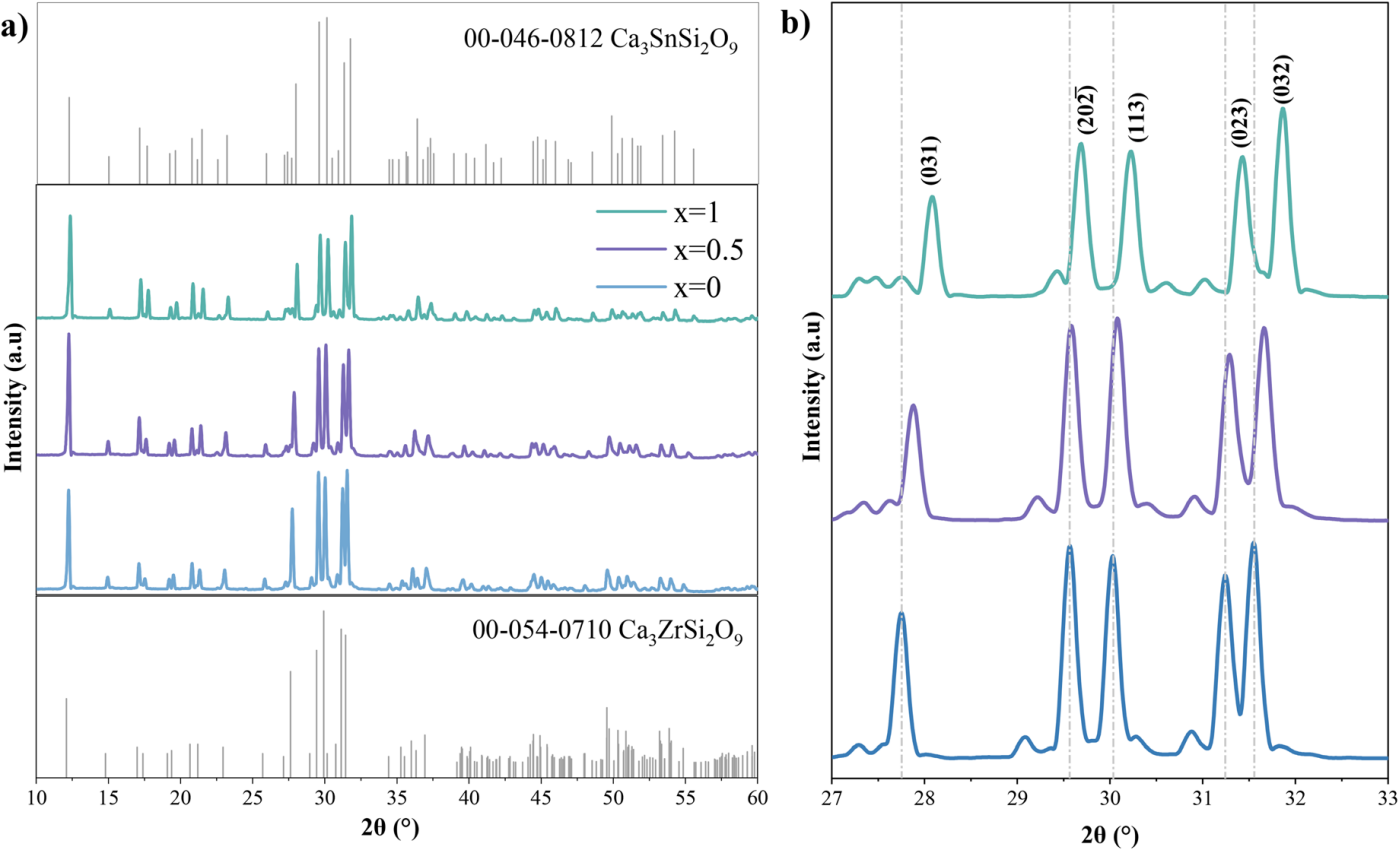
(a) Experimental XRD patterns, (b) magnified view of the main diffraction peaks (27–33°) for Ca_3_Zr_1−*x*_Sn_*x*_Si_2_O_9_ with *x* = 0, 0.50, 1.

The X-ray diffraction (XRD) patterns of the Ca_3_Zr_1−*x*_Sn_*x*_Si_2_O_9_ (*x* = 0, 0.50, and 1) samples calcined at 1430 °C are presented in Fig. 2(a). The experimental diffractograms were compared with the standard reference patterns obtained from the ICDD PDF-2 database (00-054-0710, and 00-046-0812) using the MATCH program. All reflections were indexed to the monoclinic phase with the space group *P*2_1_/*c* (No. 14), indicating the successful formation of single-phase compounds without any detectable secondary phases or impurities. This confirms the phase purity and the effectiveness of the solid-state synthesis route used.

To further analyze the effect of Sn substitution on the crystal structure, a magnified view of the most intense diffraction region (27° ≤ 2*θ* ≤ 33°) is shown in Fig. 2(b). This range corresponds mainly to the (031), (202̄), (113), (023), and (032) planes, which are sensitive to lattice variations. It is observed that with increasing Sn content (*x* = 1), the diffraction peaks gradually shift toward higher 2*θ* values. Such a shift indicates a slight contraction of the lattice parameters, in accordance with Bragg's law:^28^

1 *nλ* = 2*d* sin *θ*

where: *n* is the diffraction order, *λ* is the X-ray wavelength, *d* is the interplanar spacing, and *θ* is the Bragg diffraction angle.

According to this relation, an increase in the Bragg angle (2*θ*) corresponds to a decrease in the interplanar spacing *d*, confirming the lattice contraction.

The Rietveld refinement has been performed using Profex software (Version 5.6.1),^29^ to further validate the structural evolution upon Sn substitution. The refinement confirms the monoclinic *P*2_1_/*c* structure and provides refined lattice parameters describing the structural evolution upon Sn incorporation. Refined lattice parameters with improved accuracy are now reported.

The detailed refinement results, including lattice parameters and statistical agreement factors (*R*_wp_, *R*_exp_, *χ*^2^, and the goodness-of-fit √*χ*^2^) have been included in Table 1. The relatively low reliability factors indicate satisfactory convergence of the refinement and good quality of the structural model. The quality of the refinement is further illustrated in Fig. S1(a–d), provided in the SI, which show excellent agreement between the experimental (black line) and calculated (red line) patterns.

**Table 1:** Experimental and calculated lattice parameters, equilibrium volume, density, and corresponding deviations for Ca_3_Zr_1−*x*_Sn_*x*_Si_2_O_9_ compared with previous experimental data

		*x*	0	0.5	1
This work	Exp	*a* (Å)	7.3697	7.350	7.340
		*b* (Å)	10.1867	10.120	10.080
		*c* (Å)	10.4511	10.440	10.450
		*b* (°)	90.9379	91.0032	91.0991
		*V* (Å^3^)	784.49	776.429	773.024
		*ρ* (g cm^−3^)	3.49	3.64	3.77
		*R*_wp_	6.42%	6.83%	7.65%
		*R*_exp_	2.99%	3.06%	3.56%
		*χ* ^2^	4.61	4.98	4.62
		GoF	2.15	2.23	2.15
	Calc	*a* (Å)	7.417	7.414	7.409
		*b* (Å)	10.294	10.253	10.207
		*c* (Å)	10.489	10.501	10.514
		*b* (°)	91.097	91.096	91.152
		*V* (Å^3^)	800.782	798.188	795.090
		*ρ* (g cm^−3^)	3.414	3.539	3.668
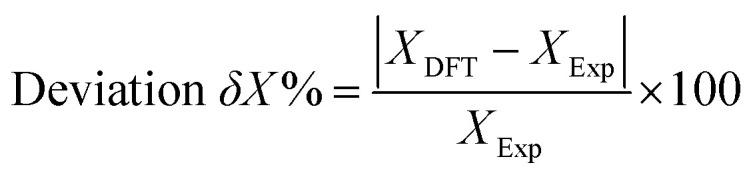		*δ*_a_ (%)	0.64	0.87	0.94
		*δ*_b_ (%)	1.05	1.31	1.26
		*δ*c (%)	0.36	0.58	0.61
		*δ*_β_ (%)	0.17	0.10	0.06
		*δ*_V_ (%)	2.08	2.80	2.85
		*δ*_ρ_ (%)	2.18	2.77	2.71
Other work	Exp^21^	*a* (Å)	7.363	7.344	7.323
		*b* (Å)	10.187	10.125	10.069
		*c* (Å)	10.453	10.442	10.430
		*V* (Å^3^)	784.02	776.44	769.06
		*ρ* (g cm^−3^)	3.49	3.64	3.79

To further investigate the distribution of Sn atoms at *x* = 0.5, both a disordered solid-solution model and several ordered configurations with explicit Sn placement on different octahedral sites were considered. The corresponding simulated XRD patterns (Fig. S2, SI) reproduce the main experimental reflections and indicate that the experimental compound is best described by a statistically disordered solid solution with an average *P*2_1_/*c* symmetry. Among the investigated ordered models, the (1, 2) configuration exhibits the closest agreement with the experimental diffraction data and was therefore selected as a representative structure for the subsequent first-principles calculations.

Following this verification, the structural optimization of the Ca_3_Zr_1−*x*_Sn_*x*_Si_2_O_9_ solid solution was carried out for compositions *x* = 0.0, 0.5, and 1.0, representing Zr-rich, equimolar, and Sn-rich phases. The optimized lattice parameters, unit cell volumes, and calculated densities are summarized in Table 1. A systematic variation in the lattice constants was observed across the studied compositions: the *a* and *b* lattice parameters exhibit a slight but consistent decrease, while the *c*-axis shows a gradual increase. The monoclinic angle *β* remains nearly constant, displaying only minor fluctuations. A notable trend is the continuous decrease of the unit cell volume as *x* increases. This effect can be rationalized by the difference in ionic radii between Zr and Sn cations: Sn^4+^ (0.69 Å) is slightly smaller than Zr^4+^ (0.72 Å) in six-fold coordination.^30^ Simultaneously, the theoretical density increases from 3.41 g cm^−3^ at *x* = 0 to 3.67 g cm^−3^ at *x* = 1.0, a trend attributed to both the volume contraction and the higher atomic mass of Sn relative to Zr. The deviations between the calculated and experimental structural parameters remain below 1.4% for the lattice constants and below 3% for the unit-cell volume, demonstrating good agreement between the DFT-optimized structures and the experimentally refined models. These structural changes highlight the progressive densification of the crystal lattice upon Sn incorporation.

To assess the energetic stability of Ca_3_Zr_1−*x*_Sn_*x*_Si_2_O_9_, cohesive energy (*E*_Coh_), formation enthalpy (Δ*H*_f_), and mixing enthalpy (Δ*H*_mix_) were calculated using the following formulas:^31^2
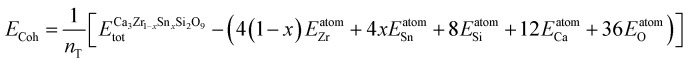
3
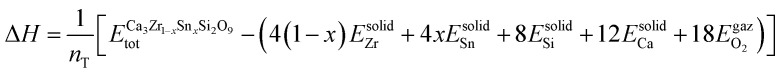
4
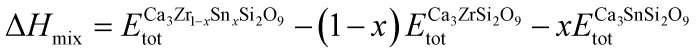
where *n*_T_ is the numbers of atoms in the Ca_3_Zr_1−*x*_Sn_*x*_Si_2_O_9_ compounds' unit-cell; 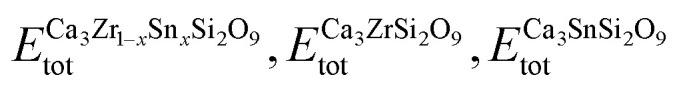, are the unit-cell's total energy; 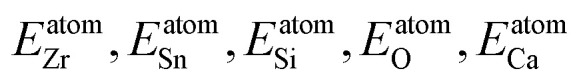 symbolizes the total energies of the isolated atoms; 
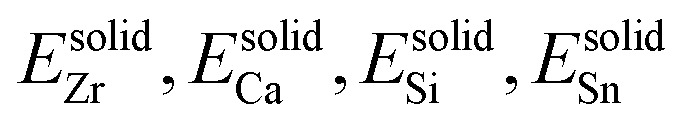
 are the total energies per atom of the solid state of the pure elements; 
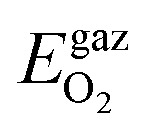
 is total energy per atom of the pure element O in its gas state.

As shown in Table 2, the cohesive energy becomes progressively less negative as Sn content increases. This trend indicates a slight reduction in average bond strength upon Sn substitution, due to weaker Sn–O interactions compared to Zr–O. Conversely, the Δ*H*_f_ decreases (more negative) across the series, indicating improved thermodynamic stability with higher Sn content. This suggests that Sn-rich compositions are more energetically favorable relative to their constituent oxides. The mixing enthalpy values are consistently negative for the intermediate composition (*x* = 0.5), confirming that the formation of the solid solution is energetically favorable. Together, these results confirm the feasibility and energetic stability of forming a continuous Ca_3_Zr_1−*x*_Sn_*x*_Si_2_O_9_ solid solution.

**Table 2:** Calculated cohesive energy (*E*_Coh_), formation enthalpy (Δ*H*_f_), and mixing enthalpy(Δ*H*_mi*x*_) of Ca_3_Zr_1−*x*_Sn_*x*_Si_2_O_9_

*x*	0	0.5	1
*E*_Coh_ (eV per atom)	−6.616	−6.312	−6.016
Δ*H*_f_ (eV per atom)	−8.846	−9.034	−9.232
Δ*H*_mix_ (eV)	0	−0.243	0

**Fig. 3:**
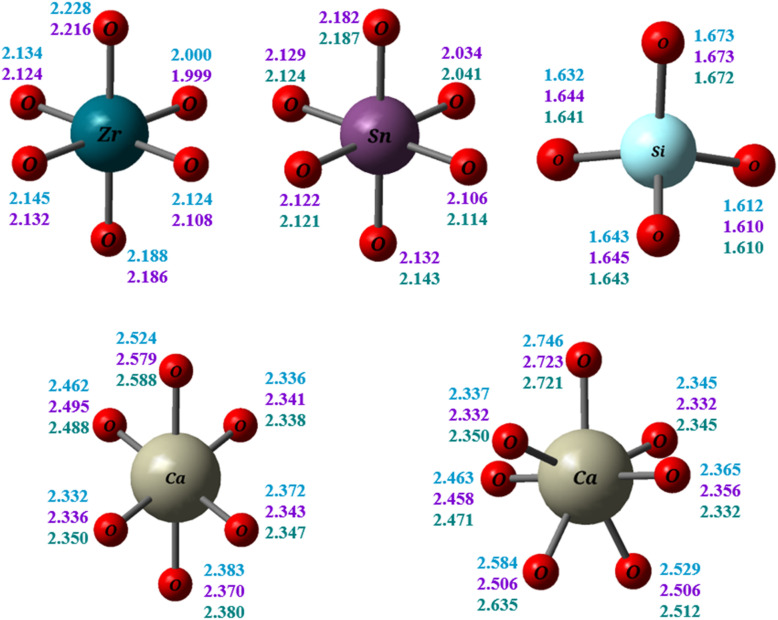
Calculated interatomic distances Ca_3_Zr_1−*x*_Sn_*x*_Si_2_O_9_ (*x* = 0, 0.50, 1).

To further probe the structural features, the optimized interatomic distances were examined (Fig. 3). The Si–O bonds remain nearly constant at ∼1.61–1.67 Å across all compositions, confirming the rigidity of the [SiO_4_] tetrahedra. In contrast, the Zr/Sn-site octahedra show a clear substitutional effect: Zr–O distances range from 2.00–2.23 Å at *x* = 0, while partial (*x* = 0.5) and full (*x* = 1) replacement by Sn yields slightly shorter Sn–O bonds (∼2.03–2.18 Å), consistent with the smaller ionic radius of Sn^4+^. This contraction of the [SnO_6_] octahedra accounts for the reduction in lattice volume discussed earlier. The Ca–O coordination shells (2.33–2.74 Å) remain more distorted but only weakly influenced by substitution, reflecting the intrinsic flexibility of the [CaO_6_]/[CaO_7_] polyhedra. Overall, substitution induces local distortions primarily around the octahedral sites, while the monoclinic symmetry and the robust 3D framework of corner-sharing polyhedra are preserved throughout the series, thereby providing a structural basis for the other properties discussed below.

### 3.2. Raman spectroscopy

**Fig. 4:**
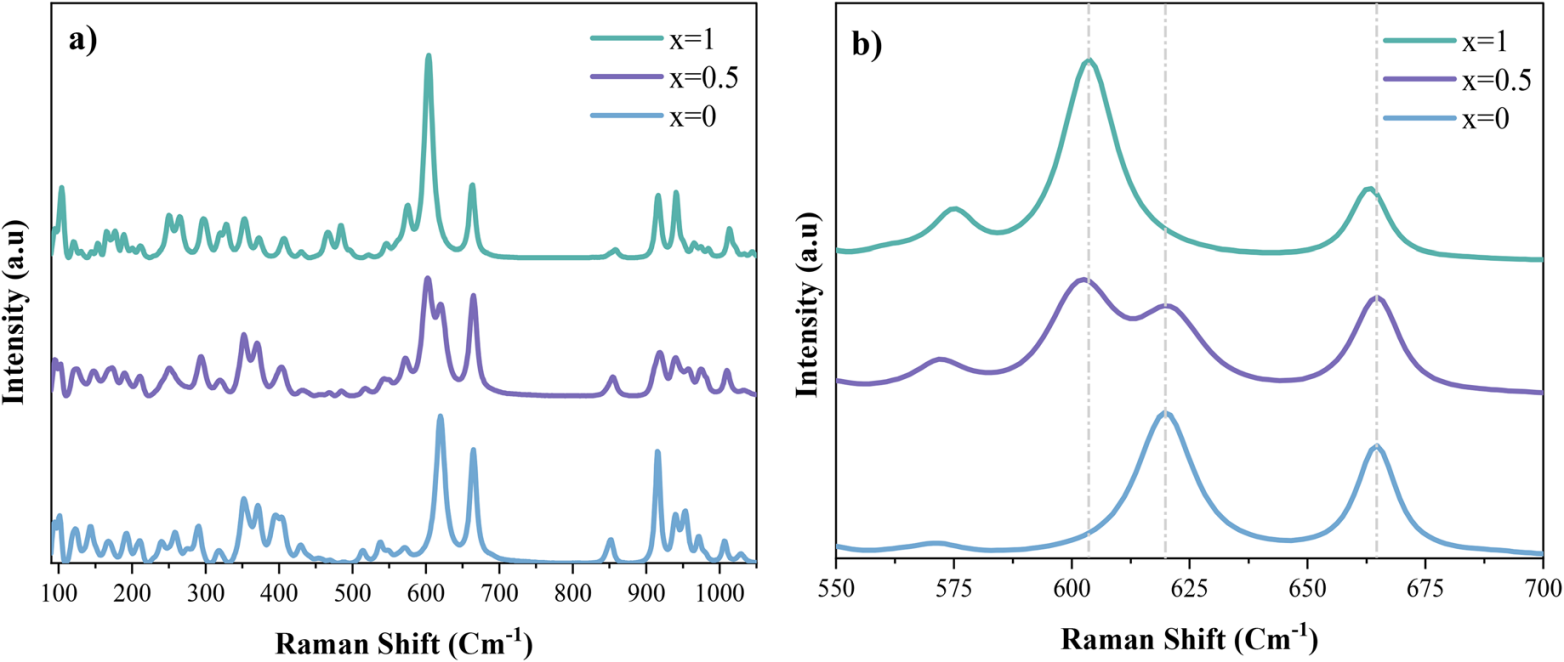
(a) Room temperature Raman spectra of Ca_3_Zr_1−*x*_Sn_*x*_Si_2_O_9_ (*x* = 0, 0.50, 1), (b) magnified view of the main peaks (550–700 cm^−1^).

Raman spectroscopy was employed to investigate the local structural environment and vibrational dynamics of the Ca_3_Zr_1−*x*_Sn_*x*_Si_2_O_9_ (*x* = 0, 0.5, and 1) ceramics. The room-temperature Raman spectra recorded in the 200–1000 cm^−1^ range are shown in Fig. 4(a). All compositions display the characteristic vibrational features of the baghdadite-type structure, consistent with earlier reports,^32^ indicating that the fundamental framework is maintained upon Sn substitution.

The observed Raman bands can be divided into three principal categories based on their vibrational origin:

• Stretching modes (s): high-frequency region (1034 cm^−1^ to 663 cm^−1^) arise from symmetric (s.s.) and antisymmetric (a.s.) stretching vibrations of Si–O bonds within Si–O–Si, Si–O–Ca, and Si–O–(Zr/Sn) linkages.

• Bending modes (b): mid-range bands (620 cm^−1^ to 353 cm^−1^) are associated with bending vibrations of Zr–O–Zr, Sn–O–Sn, Si–O–Si, O–Zr–O, and O–Si–O units.

• Rotational/low-frequency modes (r, s): modes below ∼325 cm^−1^ correspond to rotational (*r*) modes of SiO_4_ tetrahedra, as well as stretching (*s*) vibrations involving Ca–O, Si–O–Zr, and Si–O–Sn bonds.

A detailed listing of Raman mode assignments and symmetry labels is provided in the SI (Table S1).

A magnified view of the 550–700 cm^−1^ region (Fig. 4(b)) reveals a clear compositional dependence of the mid-frequency modes. The undoped sample (*x* = 0) exhibits a dominant band near ∼620 cm^−1^, characteristic of Zr–O–Zr bending vibrations. In contrast, the fully substituted composition (*x* = 1) displays a corresponding band shifted to ∼603 cm^−1^, consistent with bending vibrations of Sn–O–Sn units. The intermediate composition (*x* = 0.5) shows both contributions, with peaks at ∼620 cm^−1^ and ∼603 cm^−1^, indicating the coexistence of Zr–O–Zr and Sn–O–Sn local environments.

The red-shift and slight broadening of the Raman bands with increasing Sn content indicate lattice relaxation and local disorder caused by the substitution of Zr^4+^ by Sn^4+^ in octahedral sites. This substitution slightly modifies the metal–oxygen bond strength, resulting in softer vibrational modes. These observations confirm the successful incorporation of Sn into the Ca_3_ZrSi_2_O_9_ lattice, preserving the monoclinic baghdadite structure while introducing minor structural distortions.

### 3.3. Microstructural analysis

**Fig. 5:**
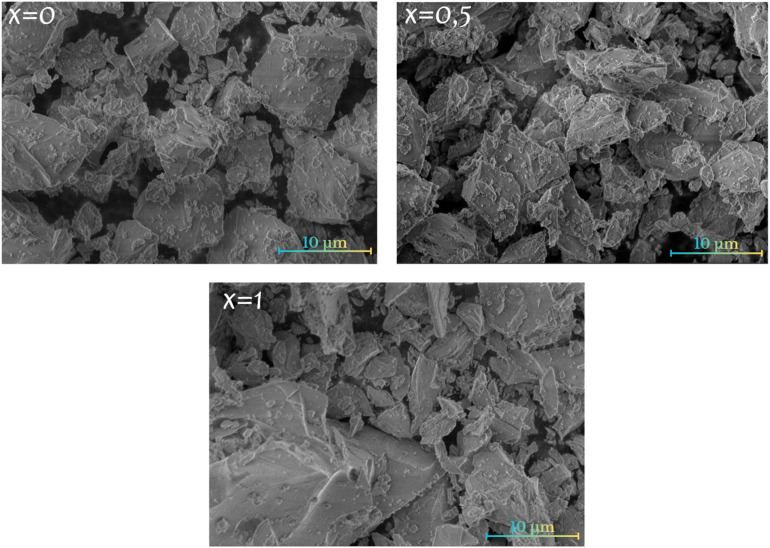
SEM images for Ca_3_Zr_1−*x*_Sn_*x*_Si_2_O_9_ powders (*x* = 0, 0.5, 1).

The morphology of Ca_3_Zr_1−*x*_Sn_*x*_Si_2_O_9_ (*x* = 0, 0.5, 1) were examined by SEM (Fig. 5). Although the overall morphology remains plate-like across the series, a progressive increase in surface roughness and micro-fragmentation is observed upon Sn substitution. This is manifested by more irregular edges, small detached flakes, and finer debris surrounding the grains, likely resulting from local lattice distortions introduced by Sn^4+^ incorporation.

Elemental composition was analyzed by EDS to verify the chemical stoichiometry. The experimental atomic percentages are in reasonable agreement with the nominal theoretical values calculated from the chemical formula Ca_3_Zr_1−*x*_Sn_*x*_Si_2_O_9_ (Table 3). The theoretical atomic percentages were determined according to:5
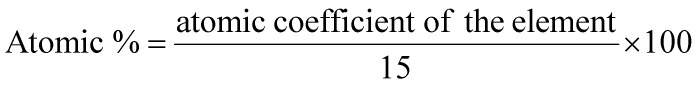
where 15 corresponds to the total number of atoms per formula unit. Minor deviations between experimental and theoretical values are attributed to the semi-quantitative nature of EDS measurements and possible surface inhomogeneities.

**Table 3:** Experimental (EDS) and nominal theoretical atomic percentages for Ca_3_Zr_1−*x*_Sn_*x*_Si_2_O_9_ (*x* = 0, 0.5, and 1) samples

Sample	Atomic percentages (%)					
		Ca	Zr	Sn	Si	O
Ca_3_ZrSi_2_O_9_	Exp	18.4	6.2	—	12.6	62.8
	Theo	20	6.7	—	13.3	60.0
Ca_3_ Zr_0.5_Sn_0.5_ Si_2_O_9_	Exp	18.2	3.1	3.0	12.0	63.7
	Theo	20	3.3	3.3	13.3	60.0
Ca_3_SnSi_2_O_9_	Exp	19.7	—	6.5	11.5	62.3
	Theo	20	—	6.7	13.3	60.0

Overall, the EDS results confirm the successful incorporation of Sn into the Ca_3_ZrSi_2_O_9_ lattice and support the formation of a continuous solid solution. Detailed spectra and quantitative data are provided in the SI (Fig. S3).

### 3.4. Electronic properties

**Fig. 6:**
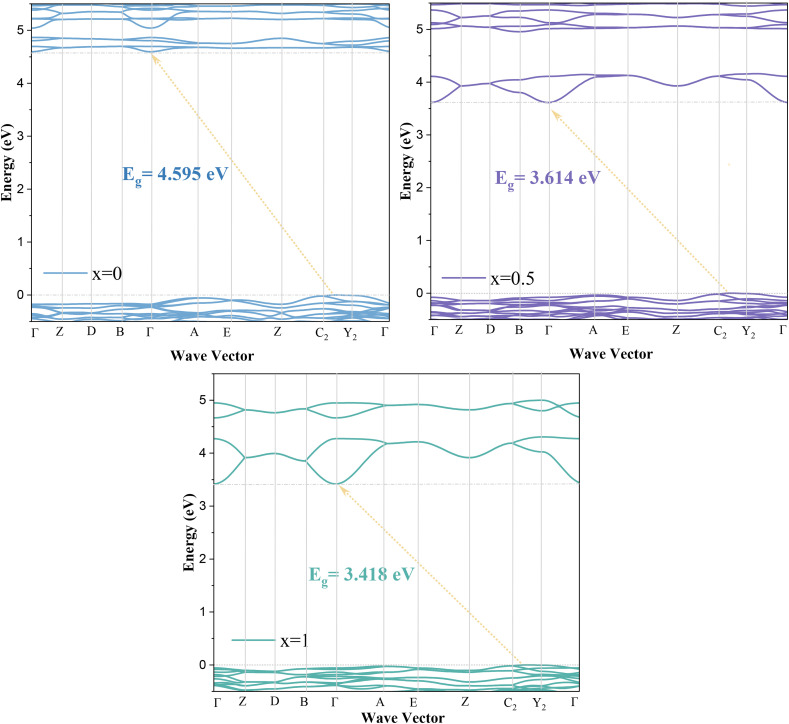
Calculated band structures for Ca_3_Zr_1−*x*_Sn_*x*_Si_2_O_9_ using GGA.

A deeper understanding of the electronic behavior of Ca_3_Zr_1−*x*_Sn_*x*_Si_2_O_9_ was obtained by analyzing the band structure and density of states. The band structure calculations confirm that all compositions exhibit an indirect band gap along the *C*_2_–*Γ* direction, with the gap magnitude decreasing upon Sn substitution. This reflects the sensitivity of the conduction band minimum to Zr-site substitution (see Fig. 6).

**Fig. 7:**
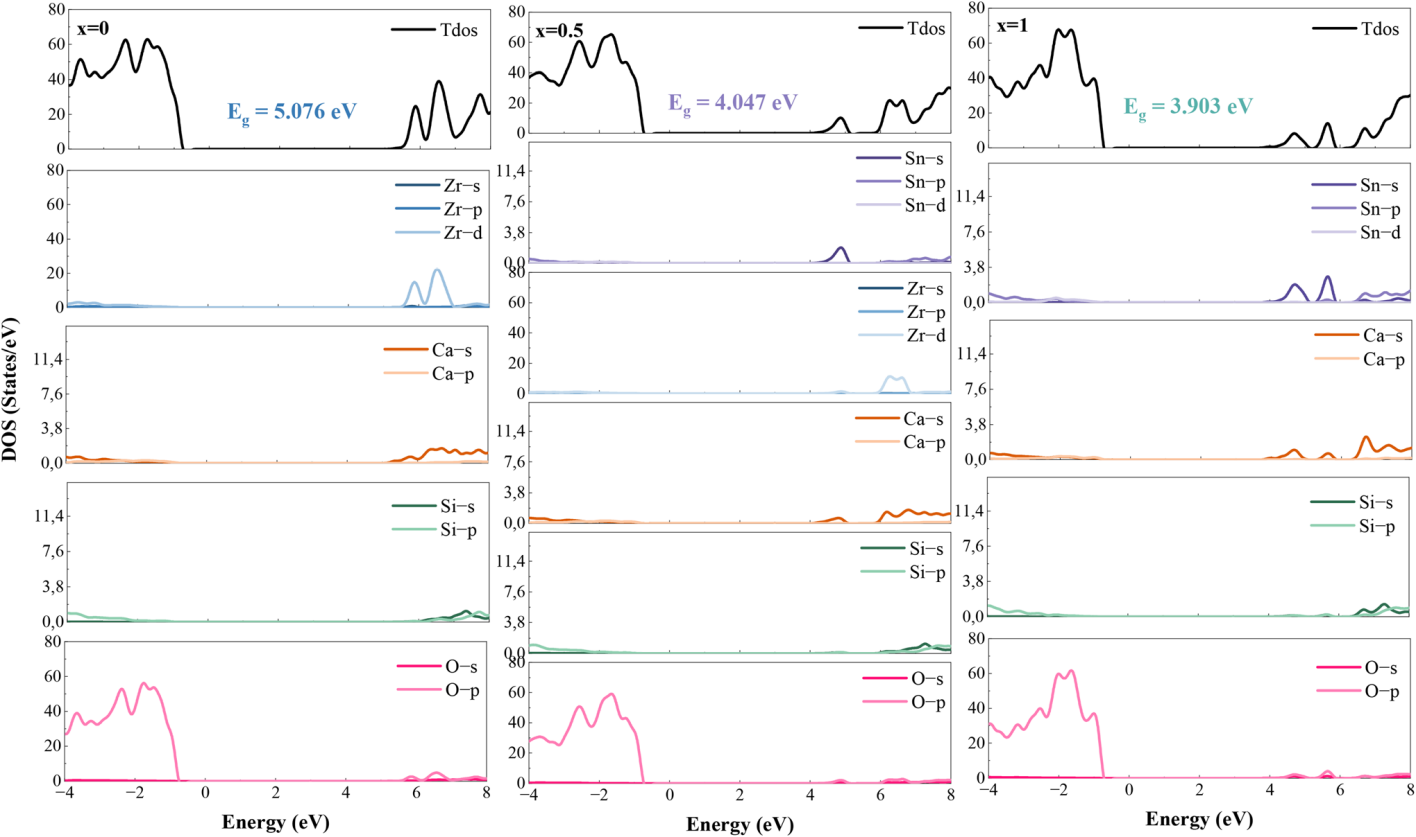
Calculated total density of states (TDOS) and projected density of states (PDOS) of Ca_3_Zr_1−*x*_Sn_*x*_Si_2_O_9_*via* HSE.

To obtain more accurate estimates, hybrid functional (HSE06) calculations were performed. These confirm the GGA-predicted compositional trends while providing corrected gap values. The total and projected density of states (TDOS/PDOS) obtained within the HSE frameworks (Fig. 7) further clarify the orbital contributions to the electronic structure. For comparison, the corresponding GGA TDOS/PDOS are provided in the SI (Fig. S4). The valence band is dominated by O–2p states, hybridized to a smaller extent with Si 3s/3p orbitals, while the conduction band edge is primarily composed of Zr–d or Sn–d states. Si contributions remain negligible near the Fermi level, consistent with the strong covalency of the [SiO_4_] tetrahedra, and Ca orbitals appear at higher energies, indicating their minor role in the electronic states near the band edges. Upon Sn substitution, the Sn-d states shift downward relative to the Zr-d states, while the stronger contribution of Sn-5s/5p orbitals near the conduction band lowers the CBM energy. This combined effect systematically reduces the band gap and modifies the conduction band dispersion.

### 3.5. Diffuse reflectance spectra (DRS)

**Fig. 8:**
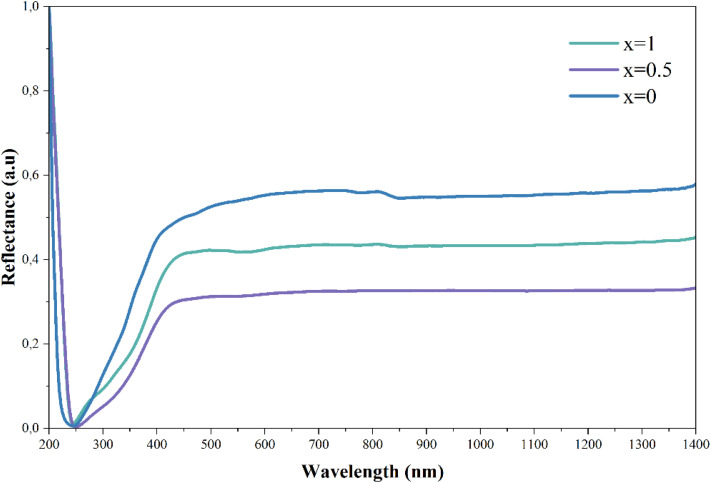
Diffuse reflectance spectra (DRS) of Ca_3_Zr_1−*x*_Sn_*x*_Si_2_O_9_ (*x* = 0, 0.5, and 1).

The diffuse reflectance spectra of Ca_3_Zr_1−*x*_Sn_*x*_Si_2_O_9_ (*x* = 0, 0.5, and 1), presented in Fig. 8, show a strong absorption edge in the ultraviolet region, characteristic of wide-band-gap oxides. For the undoped compound (*x* = 0), the main absorption band observed below 400 nm is attributed to charge-transfer transitions from O^2−^ (2p) orbitals to the empty Zr^4+^ (4d) states.

Upon Sn incorporation (*x* = 0.5 and 1), the absorption edge slightly shifts toward longer wavelengths, indicating that the optical response is affected by substitutional effects. In addition, a weak shoulder appears between 350 and 400 nm for the Sn-containing samples, which is absent in the undoped composition. This additional feature may arise from localized electronic states or charge-transfer transitions involving Sn–O bonds. Beyond approximately 450 nm, all samples display nearly constant reflectance, with a slight decrease in reflectivity as the Sn content increases, reflecting enhanced light absorption. These results demonstrate that Sn incorporation alters the optical characteristics of Ca_3_ZrSi_2_O_9_, justifying a more detailed investigation of the corresponding optical band-gap evolution, which is discussed in the following section.

#### 3.5.1. Optical bandgap determination

The optical band gap (*E*_g_) values of the Ca_3_Zr_1−*x*_Sn_*x*_Si_2_O_9_ compounds were determined from the diffuse reflectance data using the Kubelka–Munk function, defined as:

6 *F*(*R*) = (1 − *R*)^2^/2*R*

where *R* is the measured diffuse reflectance. For optically thick samples, *F*(*R*) is proportional to the absorption coefficient (*F*(*R*) ≈ *K*/*S*), assuming a constant scattering coefficient.

The absorption edge was analyzed using the Tauc relation:^33^

7 [*F*(*R*)*hν*]^*n*^ = *A*(*hν* − *E*_g_)

where *A* is a proportionality constant, *hν* is the photon energy, *E*_g_ is the optical band gap, and *n* denotes the type of electronic transition (with *n* = 1/2 for indirect and *n* = 2 for direct allowed transitions).

**Fig. 9:**
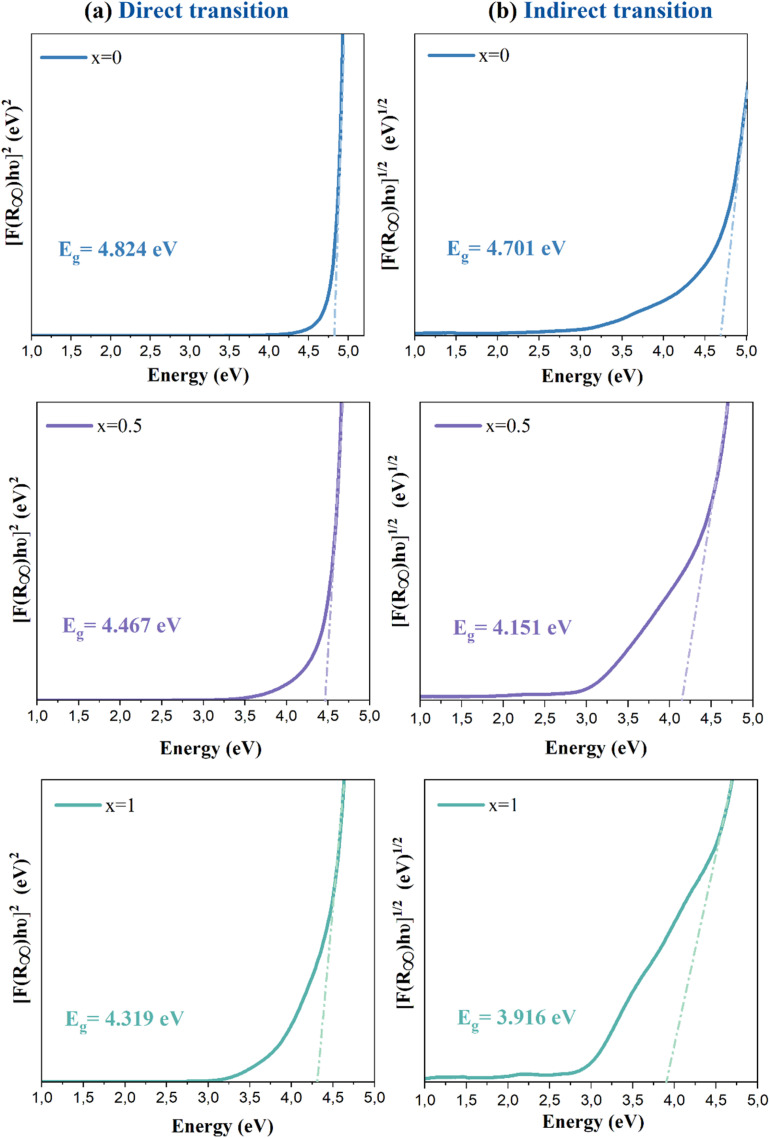
Experimental Band gap estimation from Tauc plots for Ca_3_Zr_1−*x*_Sn_*x*_Si_2_O_9_ (*x* = 0, 0.5, 1.0): (a) indirect transition (b) direct transition.

The plots of [*F*(*R*)*hν*]^*n*^*versus hν* (Fig. 9) exhibit approximately linear regions near the absorption edge, whose extrapolation to the energy axis yields the optical band gaps. The extracted values indicate that the indirect transition energies are smaller than the direct ones, which is consistent with the indirect band-gap character predicted by the DFT band-structure calculations (Table 4). The progressive red shift of the absorption edge with increasing Sn concentration indicates that Sn substitution effectively narrows the band gap. This behavior is attributed to the partial replacement of Zr^4+^ (4d^0^) by Sn^4+^ (5s^2^ 5p^2^), which modifies the conduction-band states and reduces the energy separation between the valence and conduction bands. The experimental findings are in good agreement with the calculated electronic structures, confirming that Sn incorporation tunes the optical and electronic structures of the Ca_3_Zr_1−*x*_Sn_*x*_Si_2_O_9_ compounds toward lower energy transitions.

**Table 4:** Experimental and calculated Band gap values for Ca_3_Zr_1−*x*_Sn_*x*_Si_2_O_9_ (*x* = 0, 0.5, 1.0)

*x*		*E*_gap_ (eV)		
		0	0.5	1
Exp		4.701	4.151	3.916
Calc	GGA	4.595	3.614	3.418
	HSE	5.076	4.047	3.903

### 3.6. Mechanical properties

Elastic constants, derived from first-principles calculations, provide fundamental insights into the response of a crystal under external stress and allow one to assess its mechanical stability, stiffness, ductility, and brittleness. For monoclinic Ca_3_Zr_1−*x*_Sn_*x*_Si_2_O_9_ (*x* = 0, 0.5, 1), thirteen independent elastic constants (*C*_*ij*_) were calculated to fully describe the elastic response (Table 5. The mechanical stability of all compositions was evaluated using the Born stability criteria for monoclinic systems.^34^ All computed *C*_*ij*_ values meet these requirements, confirming that our systems remain mechanically stable across the investigated compositions. The principal elastic constants show a slight decrease with increasing Sn content, reflecting a minor reduction in directional stiffness due to weaker Sn–O bonding relative to Zr–O.

**Table 5:** Calculated elastic constants of the monoclinic Ca_3_Zr_1−*x*_Sn_*x*_Si_2_O_9_ (*x* = 0, 0.5, 1)

Elastic constants *C*_*ij*_ in (GPa)			
*x*	0	0.5	1
*C*_11_	317.288	313.448	312.005
*C*_22_	274.122	271.966	274.488
*C*_33_	283.997	276.837	271.284
*C*_44_	88.416	87.397	87.845
*C*_55_	87.917	84.921	82.567
*C*_66_	98.748	96.020	95.119
*C*_12_	81.825	79.220	76.223
*C*_13_	78.309	73.182	68.801
*C*_15_	0.603	0.0005	0.269
*C*_23_	77.531	77.135	75.112
*C*_25_	0.456	0.697	0.035
*C*_35_	−0.487	0.194	2.231
*C*_46_	−0.068	−0.728	−0.212

In addition, the Cauchy's pressure^35^ (*C*_p_ = *C*_12_–*C*_44_) was employed to gain qualitative insight into the nature of atomic bonding within the solid solution. Positive *C*_p_ values are generally associated with ionic bonding character, whereas negative values suggest dominant covalent bonding. For the studied compositions, *C*_p_ values are consistently negative, with −6.59 GPa at *x* = 0, −8.18 GPa at *x* = 0.5, and −11.62 GPa at *x* = 1, confirming the covalent character of the framework. The progressive decrease of *C*_p_ across the series further suggests that Sn incorporation strengthens the covalent contribution to bonding.

To further assess the mechanical response, the macroscopic elastic moduli were derived from the calculated elastic constants using the Voigt–Reuss–Hill (VRH) approximation.^36^ The bulk modulus (*B*) reflects the resistance to volume change under hydrostatic pressure, while the shear modulus (*G*) measures the resistance to shape deformation. The Young's modulus (*E*) provides an overall measure of stiffness, and the Poisson's ratio (*ν*) describes the transverse strain response. The obtained values are summarized in Table 6.

**Table 6:** The calculated Bulk modulus (*B*_X_), Shear modulus (*G*_X_), Pugh's ratio (*B*_X_/*G*_X_), Poisson's ratio (*ν*_X_), Young's modulus (*E*_X_) in Voigt, Reuss and Voigt–Reuss–Hill approximations

	*x*	0	0.5	1
*B*_X_ (GPa)	*B*_V_	150.082	146.814	144.228
	*B*_R_	149.516	146.334	143.755
	*B*_H_	149.799	146.574	143.991
*G*_X_ (GPa)	*G*_V_	97.532	95.849	95.616
	*G*_R_	96.668	94.843	94.417
	*G*_H_	97.100	95.346	95.016
*B*_X_/*G*_X_	*B*_V_/*G*_V_	1.539	1.532	1.509
	*B*_R_/*G*_R_	1.547	1.543	1.522
	*B*_H_/*G*_H_	1.543	1.537	1.515
*ν*_X_	*ν*_V_	0.233	0.232	0.229
	*ν*_R_	0.234	0.234	0.231
	*ν*_H_	0.233	0.233	0.230
*E*_X_ (GPa)	*E*_V_	240.500	236.155	234.931
	*E*_R_	238.585	233.979	232.376
	*E*_H_	239.543	235.067	233.654

The derived moduli exhibit a gradual reduction, with the bulk modulus decreasing from about 150 to 144 GPa and the shear modulus from 97 to 95 GPa, indicating mild lattice softening across the solid solution. The *B*/*G* ratio (Pugh's criterion) and Poisson's ratio (*ν*) were employed to evaluate ductility or brittleness. A critical value of *B*/*G* ≈ 1.75 separates ductile (>1.75) from brittle (<1.75) behavior, while *ν* ≈ 0.26 serves as a secondary threshold. For Ca_3_Zr_1−*x*_Sn_*x*_Si_2_O_9_, the calculated *B*/*G* values are consistently below 1.75 and *ν* < 0.26, confirming the brittle nature characteristic of ceramics. This observation is consistent with the strong covalent bonding trend inferred from the negative Cauchy's pressure values.

To gain further insight, the acoustic wave velocities (shear *v*_s_, compressional *v*_p_, and average *v*_m_) and the Debye temperature (*Θ*_D_) ^37^were calculated using the following relations:8
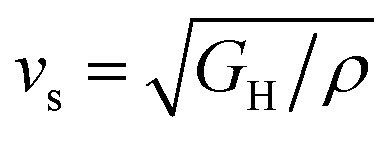
9
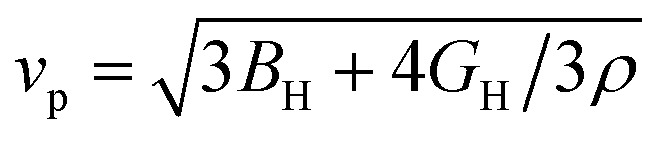
10
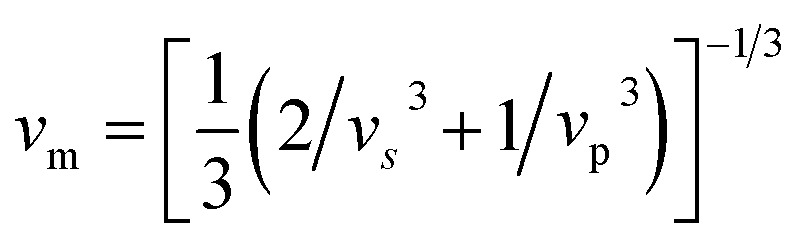

11 *Θ*_*D*_ = *h*/*k*(3*n*/4π*V*)^1/3^*v*_m_

The results (Table 7) show that all three velocities (*v*_s_, *v*_p_, *v*_m_) systematically decrease with increasing Sn concentration, consistent with the observed softening of elastic moduli. Likewise, the Debye temperature *Θ*_D_ decreases from 741.7 K (*x* = 0) to 709.0 K (*x* = 1), reflecting weaker interatomic bonding strength in Sn-substituted compositions. These findings highlight that Sn substitution in Ca_3_ZrSi_2_O_9_ reduces the overall stiffness and vibrational energy scale of the material, which may influence its thermal and mechanical reliability in applications.

**Table 7:** Wave velocities (shear *v*_s_, compressional *v*_p_, average and *v*_m_), and the Debye temperature (*Θ*_D_)

*x*	0	0.5	1
*v*_s_ (m s^−1^)	5332.893	5189.999	5085.207
*v*_p_ (m s^−1^)	9044.030	8793.368	8582.971
*v*_m_ (m s^−1^)	5902.761	5743.102	5623.976
*Θ*_D_ (K)	741.698	722.542	709.005

### 3.7. Optical properties

The investigation of optical properties provides crucial insight into the interaction of photons with matter and is essential for evaluating the potential of materials in optoelectronic and energy-related applications. Within the framework of linear response theory, the key quantity is the complex dielectric function, defined as:^38^

12 *ε*(*ω*) = *ε*_1_(*ω*) + *iε*_2_(*ω*)

where *ε*_1_(*ω*) is the real part (related to the dispersion of the electric field) and *ε*_2_(*ω*) is the imaginary part (associated with absorption due to interband transitions). The imaginary part of the dielectric function *ε*_2_(*ω*), can be directly obtained from momentum matrix elements between occupied and unoccupied states. The real part of the dielectric function *ε*_1_(*ω*), was obtained from *ε*_2_(*ω*) using the Kramers–Kronig relations.^39^

Once the dielectric function *ε*(*ω*) is determined, other important optical constants can be derived:^40^

• Refractive index *n*(*ω*): describes the speed reduction of light in the medium.13
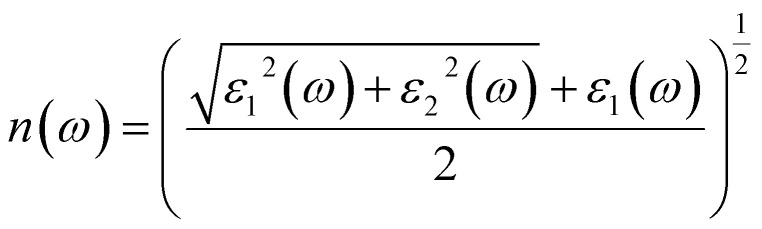


• Extinction coefficient *k*(*ω*): quantifies attenuation of light due to absorption.14
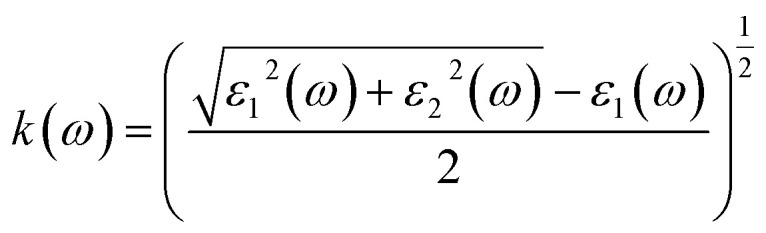


• Reflectivity *R*(*ω*): fraction of incident light reflected from the surface.15
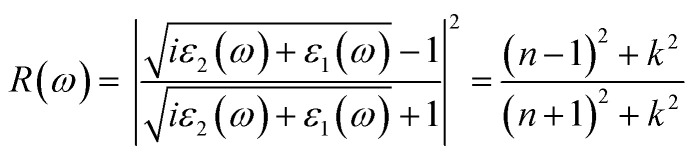


• Absorption coefficient *α*(*ω*): determines how far light can penetrate before being absorbed.16
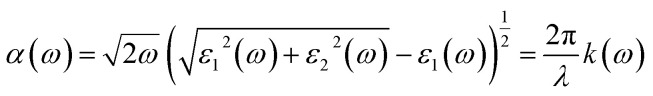


• Optical conductivity *σ*(*ω*): represents the material's response to an external electromagnetic field.17
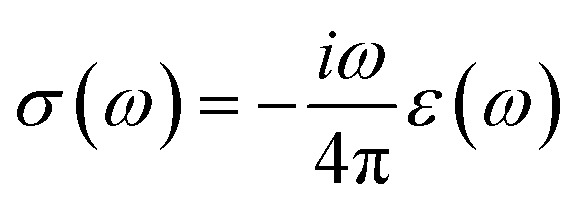


• Energy loss function *L*(*ω*): characterizes the energy loss of fast electrons traveling through the material and reveals plasma oscillations.18
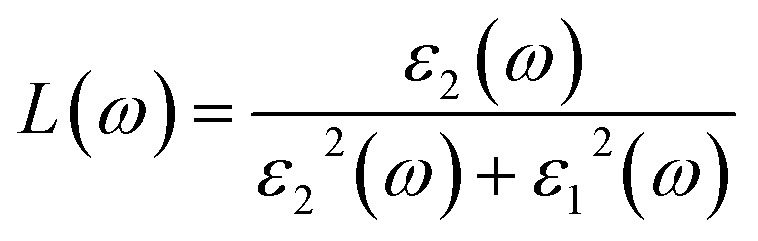


In our study, these properties have been calculated for Ca_3_Zr_1−*x*_Sn_*x*_Si_2_O_9_ compounds for photon energies up to 25 eV.

#### 3.7.1. Dielectric function *ε*(*ω*)

**Fig. 10:**
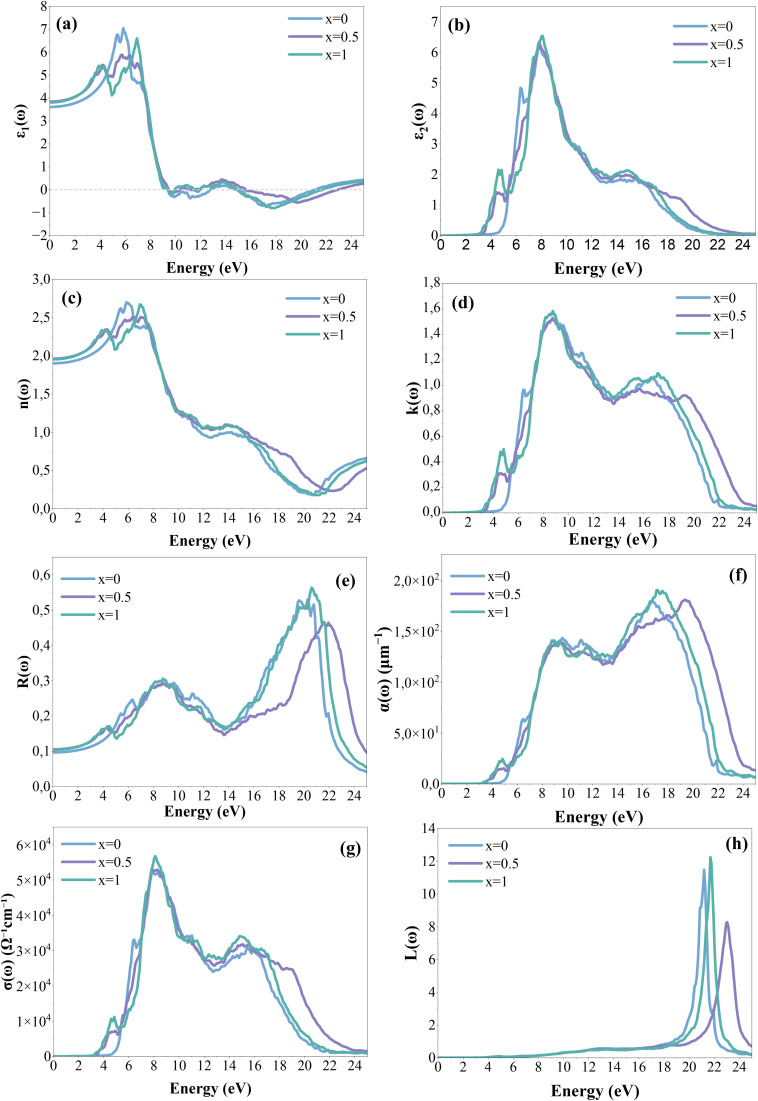
(a) Real part and (b) imaginary parts of the dielectric function, (c) refractive index (d) extinction coefficient (e) reflectivity (f) absorption coefficient (g) optical conductivity (h) energy loss function.

Fig. 10(a) shows the real part of the dielectric function, whereas Fig. 10(b) illustrates the imaginary component. The real curves *ε*_1_(*ω*) start at the static dielectric constant *ε*_1_(0), with values of 3.61 (*x* = 0), 3.85 (*x* = 0.5), and 3.81 (*x* = 1). A gradual increase in *ε*_1_(0) is observed with Sn substitution, which indicates enhanced polarizability of the system due to the contribution of Sn electronic states. After the zero-frequency limit, the curves rise steadily and reach their peak values of approximately 5.84, 6.33, 6.93 eV, for *x* = 0−1, respectively. With further increase in photon energy, *ε*_1_(*ω*) decreases and even takes negative values in the energy ranges of 9.36–12.70 eV and 14.96–22.77 eV suggesting metallic-like behavior arising from strong interband transitions. Beyond 22.77 eV, *ε*_1_(*ω*) gradually increases again and eventually returns to zero, which corresponds to the transparency region of the material at high energies.

The imaginary part *ε*_2_(*ω*), begins at a threshold energy of about 4.79 eV (*x* = 0), 3.82 eV (*x* = 0.5), and 3.61 eV (*x* = 1). These thresholds correspond to the onset of optical absorption and are directly related to the fundamental band gap of the compounds. A clear red-shift of this onset is observed with increasing Sn concentration, reflecting the band gap narrowing caused by the contribution of Sn states near the conduction band. After the threshold, *ε*_2_(*ω*) increases sharply and exhibits prominent peaks at around 7.83 eV, 7.85 eV, and 8.08 eV, which can be attributed to interband transitions from O–2p valence states to Zr–4d and Sn–5s, 5p conduction states. The peak intensity decreases slightly with increasing Sn content, while the peak positions shift toward lower photon energies, confirming the tunability of the optical absorption with chemical substitution.

#### 3.7.2. Refractive index *n*(*ω*) and extinction coefficient *k*(*ω*)

The calculated refractive index *n*(*ω*) is displayed in Fig. 10(c). These plots begins at the static value *n*(0) that increases progressively from Ca_3_ZrSi_2_O_9_ to Ca_3_SnSi_2_O_9_, in agreement with the trend in *ε*_1_(0). The curves exhibit maxima around 5.83 eV (*x* = 0), 6.38 eV (*x* = 0.5), and 6.96 eV (*x* = 1), reflecting strong dispersion in this energy range, followed by a steady decrease at higher photon energies. This behavior indicates that Sn incorporation enhances light–matter interaction at low energies, while transparency increases at higher energies.

The extinction coefficient *k*(*ω*), displayed in Fig. 10(d), shows the onset of absorption at ∼4.93 eV for *x* = 0, which shifts toward lower photon energies with 3.88 and 3.47 eV for *x* = 0, 5 and 1, respectively, consistent with the red-shifted absorption edge. Distinct peaks are observed at 8.75 eV, which correspond to interband transitions. Higher intensity in Sn-rich compositions confirms their stronger optical absorption at lower energies.

#### 3.7.3. Reflectivity *R*(*ω*) and absorption coefficient *α*(*ω*)

Fig. 10(e) illustrates the reflectivity *R*(*ω*). The spectra reveal modest reflectivity at low photon energies, followed by sharp rises near interband transition energies. The reflectivity edge is progressively shifted to hight energies as Sn concentration increases. The maximum reflectivity reaches ∼0.57 at 20.61 eV in Ca_3_SnSi_2_O_9_, which is higher than in the other compounds. This suggests that Sn substitution enhances surface reflection and modifies plasma oscillation behavior.

The absorption coefficient *α*(*ω*) is shown in Fig. 10(f), clearly exhibits the absorption onset at 5.04 eV for Ca_3_ZrSi_2_O_9_ and shifts to 3.85 eV for Ca_3_SnSi_2_O_9_, confirming a red-shift with Sn doping. Multiple absorption peaks are observed at 15–20 eV region, associated with optical transitions from O–2p states in the valence band to Zr–4d/Sn–5d states in the conduction band.

#### 3.7.4. Optical conductivity *σ*(*ω*) and energy loss function *L*(*ω*)

Fig. 10(g) depicts the optical conductivity *σ*(*ω*), which starts increasing from the respective absorption edges. Peaks appear at photon energies corresponding to those in *ε*_2_(*ω*) and *α*(*ω*), confirming that conductivity is directly governed by electronic transitions. The conductivity magnitude is higher for Sn-rich compounds, indicating that Sn incorporation enhances charge carrier excitation and electronic transport under incident photons.

Finally, the energy loss function *L*(*ω*), shown in Fig. 10(h), exhibits pronounced peaks at approximately 21.21 eV (*x* = 0) eV, 23.04 eV (*x* = 0.5), 21.73 eV (*x* = 1), corresponding to plasma resonance frequencies (plasma frequency *ω*_p_). These peaks shift slightly toward higher energies with Sn substitution, which can be attributed to increased free-carrier contribution and dielectric screening effects.

### 3.8. Band-edge alignment and photocatalytic response

To evaluate the photocatalytic potential of Ca_3_Zr_1−*x*_Sn_*x*_Si_2_O_9,_ the calculated conduction band minimum (CBM) and valence band maximum (VBM) were aligned with respect to the normal hydrogen electrode (NHE) at pH = 0. For photocatalytic water splitting, the CBM must be more negative than the H^+^/H_2_ reduction potential (0 eV *vs.* NHE), while the VBM must be below (more positive than) the O_2_/H_2_O oxidation potential (+1.23 eV *vs.* NHE). The positions of CBM and VBM can be theoretically predicted using the Mulliken electronegativity approach,^41,42^ expressed as:

19 *E*_CBM_ = *χ* − *E*_e_ + 0.5 *E*_g_

20 *E*_VBM_ = *E*_CBM_ + *E*_g_

21
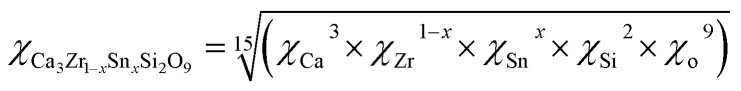
where *χ* is the absolute electronegativity of the compound (geometric mean of constituent atoms), *χ*_Ca_, *χ*_Zr_, *χ*_Sn_, *χ*_Si_, and *χ*_O_ indicate the absolute electronegativities of Ca, Zr, Sn, Si, and O elements, respectively, (*E*_e_) is the energy of free electrons on the hydrogen scale (≈4.44 eV), and (*E*_g_) is the calculated band gap. This method provides a reliable estimate of band edge positions relative to the normal hydrogen electrode (NHE).

**Fig. 11:**
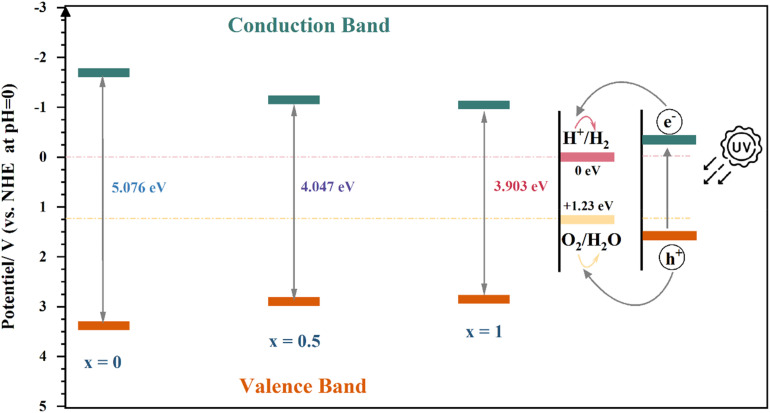
The calculated band-edge positions adopted from Mulliken electronegativity approach for Ca_3_Zr_1−*x*_Sn_*x*_Si_2_O_9_, and Potential photocatalytic mechanism.

As shown in Fig. 11, Band-edge alignment calculations show that for all compositions the CBM lies at a sufficiently negative potential relative to H^+^/H_2_ and the VBM lies at a sufficiently positive potential relative to O_2_/H_2_O, indicating that overall water splitting is thermodynamically allowed. The calculated band-edge positions reveal that Ca_3_ZrSi_2_O_9_ (*x* = 0) with the widest band gap (5.076 eV) possesses the strongest thermodynamic driving force for water splitting, as its conduction and valence bands straddle the H^+^/H_2_ and O_2_/H_2_O potentials with the largest margin. However, the large gap requires high-energy photons for excitation. With progressive Sn substitution, the band gap decreases to 4.047 eV (*x* = 0.5) and 3.903 eV (*x* = 1.0). This narrowing reduces the thermodynamic margin but improves the photoexcitation efficiency under UV irradiation, since lower-energy UV photons can be utilized. Therefore, while the *x* = 0 composition is the most favorable from a purely thermodynamic viewpoint, the *x* = 1.0 sample is more practical for photocatalytic water splitting under UV light due to its reduced band gap and enhanced photon absorption.

### 3.9. Thermoelectric properties

Thermoelectric properties provide valuable insight into the efficiency of materials in converting heat into electrical energy and *vice versa*. The key parameters that govern thermoelectric performance are: the Seebeck coefficient (*S*), which measures the induced voltage in response to a temperature gradient; the electrical conductivity (*σ*/*τ*), which reflects the ability of charge carriers to transport electricity; the electronic thermal conductivity (*κ*_e_/*τ*), representing the contribution of carriers to heat transport; and the power factor (PF = *S*^2^ × *σ*/*τ*), which serves as an important figure of merit for evaluating the potential of a material for thermoelectric applications. In the present work, we have systematically investigated these thermoelectric parameters for Ca_3_Zr_1−*x*_Sn_*x*_Si_2_O_9_ (*x* = 0, 0.5, 1) as functions of both chemical potential and temperature in the range of 300–1500 K.

#### 3.9.1. Thermoelectric properties as a function of chemical potential

**Fig. 12:**
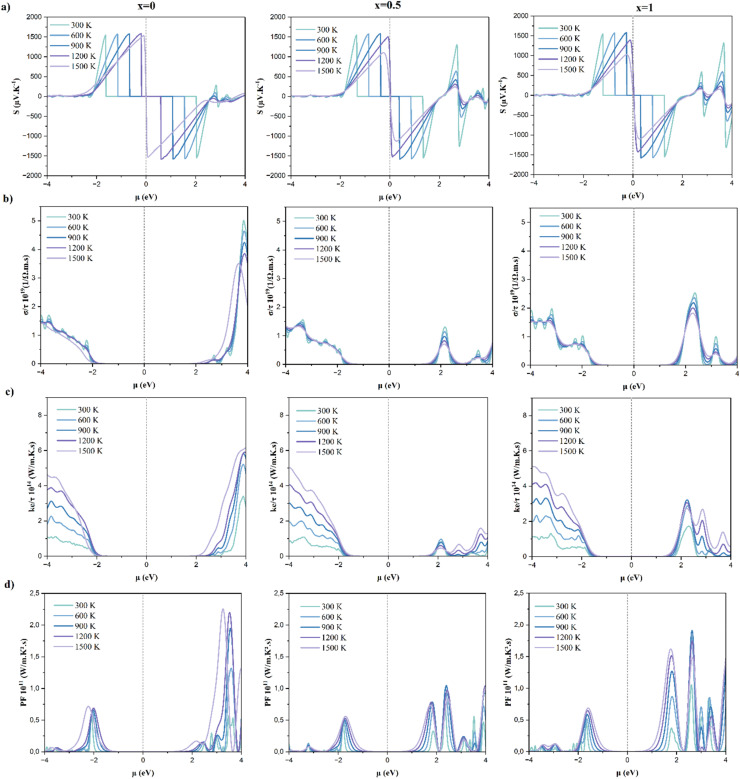
(a) Seebeck coefficient, (b) electrical conductivity, (c) electronic thermal conductivity, and (d) power factor *versus* chemical potential.

The Seebeck coefficient as a function of chemical potential at different temperatures is presented in Fig. 12(a). In the negative *µ* region, *S* attains large positive values, characteristic of p-type conduction, while in the positive *µ* region, it becomes negative, confirming n-type behavior. This bipolar nature reflects the coexistence of electron and hole contributions, and suggests that tuning the chemical potential (*via* doping or carrier engineering) allows one to control the conduction type. At lower temperatures (300–900 K), *S* maintains a comparatively constant maximum value of about 1590 µV K^−1^ for all compositions, reflecting stable carrier response in this range. Beyond 900 K, however, a gradual decline is observed, and at 1500 K the maximum Seebeck coefficients reduce to approximately 1540.85 µV K^−1^ for *x* = 0, 1105.87 µV K^−1^ for *x* = 0.5, and 1026.47 µV K^−1^ for *x* = 1. This decrease at elevated temperatures is attributed to increased intrinsic carrier excitation, which lowers the net thermopower. Notably, Sn substitution influences the rate of this decline, as increasing Sn content systematically reduces the Seebeck response, suggesting that Sn incorporation increases carrier concentration and thus weakens the thermopower.

The electrical conductivity per relaxation time (*σ*/*τ*) is presented in Fig. 12(b) in the chemical potential range of −5.0 eV to 5.0 eV at different temperatures. In the chemical range of −1.0 eV to 1.0 eV, the conductivity is nearly zero for all compositions, reflecting the lack of available carriers in this range. Beyond this interval, *σ*/*τ* begins to increase sharply on both sides of the chemical potential axis, with the onset occurring at approximately 2.05 eV for *x* = 0, 1.42 eV for *x* = 0.5, and 1.25 eV for *x* = 1 in the positive *µ* region. The conductivity values are significantly higher in the n-type region (*µ* > 0) compared to the p-type (*µ* < 0), indicating the greater mobility and availability of electron carriers relative to holes. Among the studied compositions, the *x* = 0 compound (Ca_3_ZrSi_2_O_9_) exhibits the highest maximum conductivity, followed by *x* = 1 (Ca_3_SnSi_2_O_9_), while the doped composition (*x* = 0.5) shows the lowest values. This behavior confirms that Sn substitution reduces electrical transport efficiency, consistent with increased scattering and reduced carrier mobility. Moreover, the overall *σ*/*τ* shows weak sensitivity to temperature compared with the Seebeck coefficient, this weak temperature dependence suggests that carrier concentration (determined by *µ* and composition) is the main factor governing conductivity, while thermal activation plays only a secondary role.

The electronic thermal conductivity (*κ*_e_/*τ*) (Fig. 12(c)) shows a trend similar to *σ*/*τ*, remaining negligible near the mid-gap and increasing as carriers are introduced, highlighting its carrier-dependent nature. The n-type region exhibits comparatively larger values, confirming the dominant role of electrons in heat transport. In contrast to electrical conductivity, *κ*_e_/*τ* is more sensitive to temperature. Its magnitude steadily increases with rising temperature.

Similar to the previous parameters, PF (Fig. 12(d)) is larger in the n-type region and rises with temperature. At 1500 K, the power factor reaches its maximum, following the order *x* = 0 > *x* = 1 > *x* = 0.5. This confirms that Sn substitution lowers the thermoelectric efficiency compared to the pristine compound.

#### 3.9.2. Thermoelectric properties as a function of temperature

**Fig. 13:**
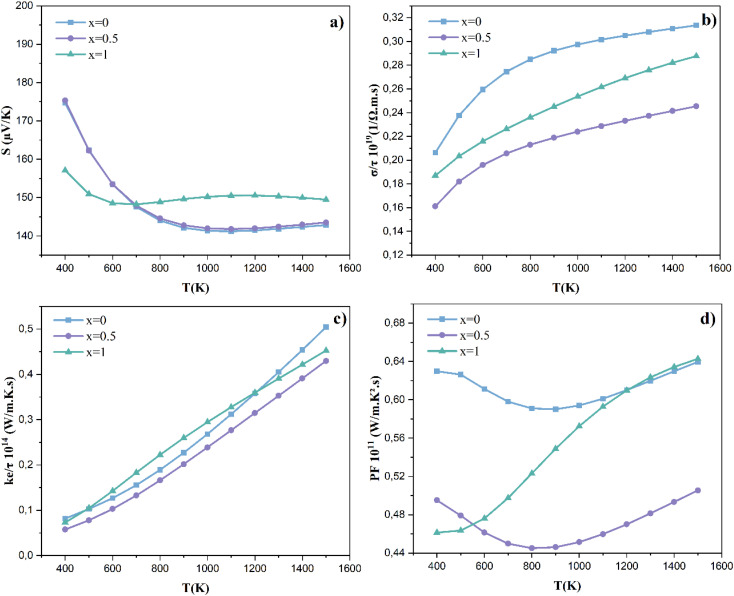
(a) Seebeck coefficient, (b) electrical conductivity, (c) electronic thermal conductivity, and (d) power factor *versus* temperature.

The temperature-dependent Seebeck coefficient is shown in Fig. 13(a). For *x* = 0 and *x* = 0.5, *S* decreases steadily with rising temperature up to ∼1000 K and then stabilizes, indicating a relatively constant carrier response at higher temperatures. In contrast, *x* = 1 composition shows a different trend: *S* drops between 400–700 K but slightly increases thereafter, suggesting a distinct carrier transport mechanism. The electrical conductivity (Fig. 13(b)) exhibits the opposite behavior, rising continuously with temperature for all compositions, with *σ*/*τ* following the order *x* = 0 > *x* = 1 > *x* = 0.5. A similar temperature-driven increase is observed for the electronic thermal conductivity (Fig. 13(c)), consistent with its carrier-dependent nature. The evolution of the power factor (Fig. 13(d)) shows a more complex behavior: at low temperatures, PF is higher for *x* = 0 and *x* = 0.5, but both exhibit a decline up to about 900 K, after which PF begins to rise again. Conversely, the *x* = 1 compound increases continuously with temperature, surpassing the other compositions above 1200 K, where it attains the highest PF values. This crossover underscores the distinct role of Sn substitution at elevated temperatures, in contrast to its unfavorable effect at low and intermediate ranges.

## 4. Conclusion

Combined experimental and theoretical investigation was conducted on Ca_3_Zr_1−*x*_Sn_*x*_Si_2_O_9_ (*x* = 0, 0.5, 1) to assess the impact of Sn substitution on structural and functional properties. XRD analysis confirmed the formation of single-phase monoclinic *P*2_1_/*c* compounds for all compositions, with peak shifts indicating lattice contraction consistent with Sn^4+^ incorporation. Raman spectroscopy further verified the preservation of the baghdadite framework, while mode shifts and broadening reflected local structural distortions introduced by Sn. SEM observations revealed similar irregular plate-like morphologies across all samples, and EDS confirmed compositional homogeneity.

UV-vis diffuse reflectance showed a clear band-gap reduction with increasing Sn content, which agrees well with first-principles calculations. DFT results also reproduced the structural trends, confirmed mechanical stability, and showed that Sn substitution slightly reduces stiffness due to weaker Sn–O bonding. Electronic structure calculations predicted progressive band-gap narrowing and favorable band-edge alignment with respect to redox potentials, suggesting potential suitability for photocatalytic water splitting. Thermoelectric transport simulations predicted composition-dependent improvements in Seebeck coefficient and power factor at elevated temperatures for Sn-rich compositions.

Overall, the combined results demonstrate that Ca_3_Zr_1−*x*_Sn_*x*_Si_2_O_9_ retains structural stability while offering tunable electronic and optical properties. The predicted electronic structure and transport behavior suggest that Sn substitution may enhance the potential of these materials for UV-responsive photocatalysis and high-temperature thermoelectric applications. However, experimental validation of photocatalytic activity and thermoelectric performance will be necessary to fully confirm these theoretical predictions.

## Author contributions

Oumnia Racha Selmi: writing – review & editing, validation, software, investigation, formal analysis, data curation, conceptualization, writing – original draft, visualization, methodology. Rachid Makhloufi: writing – review and editing, validation, supervision, project administration, methodology, investigation, formal analysis. Rania Charif: methodology, formal analysis. Validation. Ali Ismael: investigation, writing – review and editing, validation. Taha Abdel Mohaymen Taha: investigation, writing – review and editing, validation.

## Conflicts of interest

There are no conflicts to declare.

## Supplementary Material

RA-016-D6RA02396G-s001

## Data Availability

The data that support the findings of this study are available from the corresponding author upon reasonable request. Supplementary information (SI): Rietveld refinement plots (Fig. S1), simulated XRD patterns for ordered and disordered models (Fig. S2), EDS spectra (Fig. S3), GGA TDOS/PDOS plots (Fig. S4), and Raman mode assignments (Table S1). See DOI: https://doi.org/10.1039/d6ra02396g.

## References

[cit1] Dawood A. E. (2017). *et al.*, Calcium silicate-based cements: composition, properties, and clinical applications. J. Invest. Clin. Dent..

[cit2] Felipe-Sesé M., Eliche-Quesada D., Corpas-Iglesias F. A. (2011). The use of solid residues derived from different industrial activities to obtain calcium silicates for use as insulating construction materials. Ceram. Int..

[cit3] Bansal N. (2023). *et al.*, Alkali field strength effects on optical, dielectric, and conducting properties of calcium borosilicate glasses. Ceram. Int..

[cit4] No Y. J., Li J. J., Zreiqat H. (2017). Doped Calcium Silicate Ceramics: A New Class of Candidates for Synthetic Bone Substitutes. Materials.

[cit5] Zhihua L. (2023). *et al.*, Advances in the use of calcium silicate-based materials in bone tissue engineering. Ceram. Int..

[cit6] Aarzoo Z., Sayyed A. M. P., Patil S. P., Sonawane J. P., Bhavsar A. C., Quazi M. A. (2025). Metal Oxide Doped Calcium Silicate Material Having Biomedical Application. Int. J. Environ. Sci..

[cit7] Zhongyang L. (2024). *et al.*, Effect of alumina on formation mechanism and structure characteristic of sodium calcium silicate compounds with different stoichiometric coefficients. Ceram. Int..

[cit8] Liu J. (2013). *et al.*, Calcium–magnesium–aluminosilicate corrosion behaviors of rare-earth disilicates at 1400 °C. J. Eur. Ceram. Soc..

[cit9] Sun L. (2020). *et al.*, High temperature corrosion of (Er_0.25_Tm_0.25_Yb_0.25_Lu_0.25_)_2_Si_2_O_7_ environmental barrier coating material subjected to water vapor and molten calcium–magnesium–aluminosilicate (CMAS). Corros. Sci..

[cit10] Wei F. (2023). *et al.*, A systematic analysis of the calcium-magnesium-aluminosilicate corrosion behavior of high-entropy (5Re_0.2_)_2_Si_2_O_7_ materials. Corros. Sci..

[cit11] Youness R. A., Tag El-deen D. M., Taha M. A. (2022). A Review on Calcium Silicate Ceramics: Properties, Limitations, and Solutions for Their Use in Biomedical Applications. Silicon.

[cit12] Zuo Y. (2014). *et al.*, Enhanced luminescent properties of Ca_3−x_Tb_x_ ZrSi_2_O_9+(x-y)/2_ phosphors by Al^3+^ doping into the Zr^4+^ site in the host lattice. J. Lumin..

[cit13] Schumacher T. C. (2015). *et al.*, Synthesis and mechanical evaluation of Sr-doped calcium-zirconium-silicate (baghdadite) and its impact on osteoblast cell proliferation and ALP activity. Biomed. Mater..

[cit14] Pan Y. (2021). *et al.*, Research on Thermal Stability and Properties of Ca_3_ZrSi_2_O_9_ as Potential T/EBC Materials. Coatings.

[cit15] Bakhsheshi-Rad H. R. (2017). *et al.*, Synthesis of a novel nanostructured zinc oxide/baghdadite coating on Mg alloy for biomedical application: In-vitro degradation behavior and antibacterial activities. Ceram. Int..

[cit16] Hossein J., Bengi Y., Zafer E. (2020). Calcium zirconium silicate (baghdadite) ceramic as a biomaterial. Ceram. Int..

[cit17] Mehrafzoon S., Hassanzadeh-Tabrizi S. A., Bigham A. (2018). Synthesis of nanoporous Baghdadite by a modified sol-gel method and its structural and controlled release properties. Ceram. Int..

[cit18] Schumacher T. C. (2014). *et al.*, Mechanical evaluation of calcium-zirconium-silicate (baghdadite) obtained by a direct solid-state synthesis route. J. Mech. Behav. Biomed. Mater..

[cit19] Yu X. (2014). *et al.*, Improvement of the energy transfer from Ca_3_SnSi_2_O_9_ host to rare-earth ions with the assistance of oxygen vacancies. RSC Adv..

[cit20] Wu S. P. (2012). *et al.*, Synthesis and microwave dielectric properties of Ca_3_SnSi_2_O_9_ ceramics. J. Alloys Compd..

[cit21] Kan A., Ogawa H., Ohsato H. (2007). Synthesis and Crystal Structure–Microwave Dielectric Property Relations in Sn-Substituted Ca_3_(Zr_1-x_Sn_x_)Si_2_O_9_ Solid Solutions with Cuspidine Structure. Jpn. J. Appl. Phys..

[cit22] Giannozzi P. (2009). *et al.*, QUANTUM ESPRESSO: a modular and open-source software project for quantum simulations of materials. J. Phys.: Condens. Matter.

[cit23] John J. P., Burke K., Ernzerhof M. (1996). Generalized Gradient Approximation Made Simple. Am. Phys. Soc..

[cit24] Blochl P. E. (1994). Projector augmented-wave method. Phys. Rev. B: Condens. Matter.

[cit25] Heyd J., Scuseria G. E. (2004). Efficient hybrid density functional calculations in solids: assessment of the Heyd-Scuseria-Ernzerhof screened Coulomb hybrid functional. J. Phys. Chem..

[cit26] Chadi D. J. (1977). Special points for Brillouin-zone integrations. Phys. Rev. B: Condens. Matter Mater. Phys..

[cit27] Madsen G. K. H., Carrete J., Verstraete M. J. (2018). BoltzTraP2, a program for interpolating band structures and calculating semi-classical transport coefficients. Comput. Phys. Commun..

[cit28] Bragg W. H., Bragg W. L. (1997). The reflection of X-rays by crystals. Proc. R. Soc. Lond. - Ser. A Contain. Pap. a Math. Phys. Character.

[cit29] Doebelin N., Kleeberg R. (2015). Profex: a graphical user interface for the Rietveld refinement program BGMN. J. Appl. Crystallogr..

[cit30] Reddy N. N. K. (2017). *et al.*, Zr-doped SnO_2_ thin films synthesized by spray pyrolysis technique for barrier layers in solar cells. Appl. Phys. A.

[cit31] Selmi O. R., Makhloufi R., Charif R. (2025). Effect of Hf-substitution in tetragonal Zr_1-x_Hf_x_SiO_4_ ceramics: A thorough DFT investigation of structural, stability, electronic, optical, and thermoelectric properties. Phys. B.

[cit32] Dul K. (2015). *et al.*, Vibrational spectra of a baghdadite synthetic analogue. Vib. Spectrosc..

[cit33] Klein J. (2023). *et al.*, Limitations of the Tauc Plot Method. Adv. Funct. Mater..

[cit34] Liu Q.-J. (2014). *et al.*, Structural, electronic, optical, elastic properties and Born effective charges of monoclinic HfO_2_ from first-principles calculations. Chin. Phys. B.

[cit35] Al-Qaisi S. (2017). *et al.*, Structural, elastic, mechanical and thermodynamic properties of Terbium oxide: First-principles investigations. Results Phys..

[cit36] Zuo L., Humbert M., Esling C. (1992). Elastic Properties of Polycrystals in the Voigt-Reuss-Hill Approximation. J. Appl. Crystallogr..

[cit37] Noor N. A. (2018). *et al.*, Ab-initio study of thermodynamic stability, thermoelectric and optical properties of perovskites ATiO_3_ (A=Pb, Sn). J. Solid State Chem..

[cit38] Fox M. , Optical Properties of Solids, Oxford university press, 2010, Vol. 3

[cit39] Nye J. F. , Physical Properties of Crystals: Their Representation by Tensors and Matrices, Clarendon Press, 1985

[cit40] Wang H. (2020). *et al.*, First principles studies of electronic, mechanical and optical properties of Cr-doped cubic ZrO_2_. Chem. Phys..

[cit41] Pearson R. G. (1988). Absolute Electronegativity and Hardness: Application to Inorganic Chemistry. Inorg. Chem..

[cit42] Bouzaid A., Ziat Y., Belkhanchi H. (2025). Photocatalytic Optimization of ATiO_3_ Codoped with Se/Zr: A DFT Study for Hydrogen Production. Materials.

