# Supplementary material for: First-principles investigation of optoelectronic, thermoelectric, and photocatalytic properties of Ca3Zr1−xSnxSi2O9
Source: RSC Adv. 2026 Jul 2;16(34):32972–89. doi: 10.1039/d6ra02396g (PMC13326590; doi:10.1039/d6ra02396g)
Supplement: RA-016-D6RA02396G-s001 [file RA-016-D6RA02396G-s001.pdf]

Supplementary Information (SI) for RSC Advances.
This journal is © The Royal Society of Chemistry 2026

Supplementary Information

First-Principles Investigation of Optoelectronic, Thermoelectric, and Photocatalytic Properties of $Ca_3Zr_{1-x}Sn_xSi_2O_9$

Oumnia Racha Selmi[a,*], Rachid Makhloufi[a,*], Rania Charif [a], Ali Ismael[b,*], Taha Abdel Mohaymen Taha[c,*]

[a] Laboratory of Applied Chemistry (LCA), University of Biskra, PO Box 145, 07000 Biskra, Algeria.

[b] Physics Department, Lancaster University, Lancaster, LA1 4YB, UK.

[c] Physics and Engineering Mathematics Department, Faculty of Electronic Engineering, Menoufia University, Menouf, 32952, Egypt.

[*] Corresponding authors: oumniaracha.selmi@univ−biskra.dz (O. R. Selmi), r.makhloufi@univ−biskra.dz (R. Makhloufi), k.ismael@lancaster.ac.uk (A. Ismael), taha.hemida@yahoo.com (T.A. Mohaymen Taha).

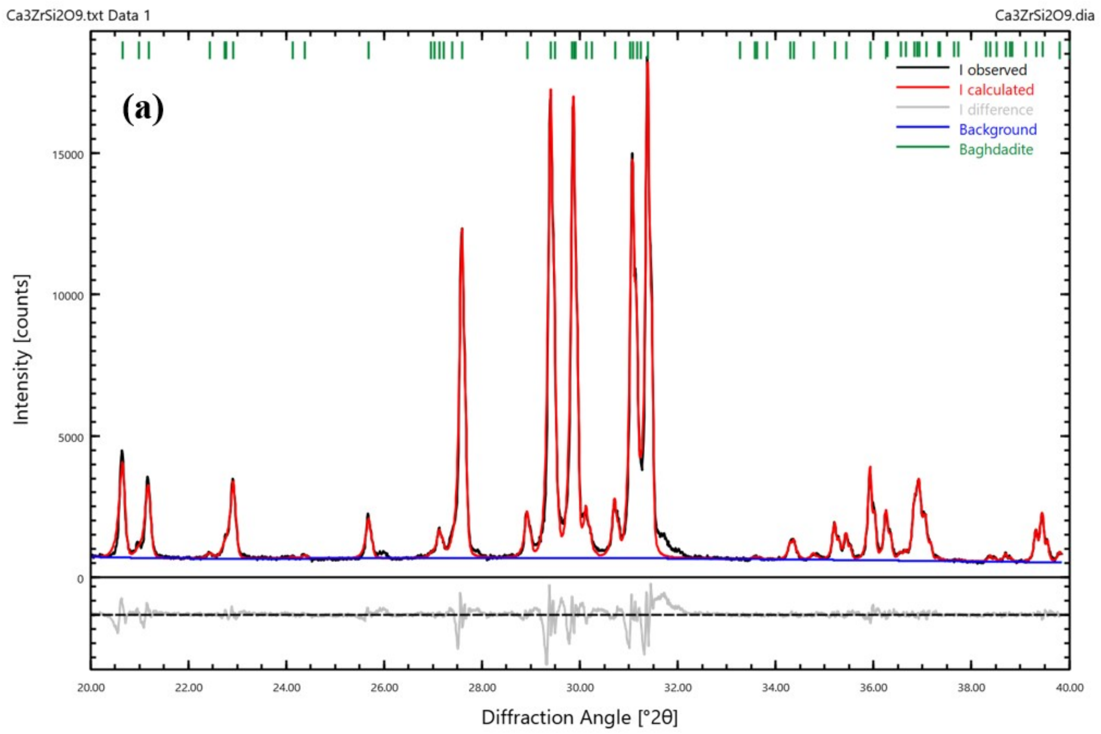
Ca3ZrSi2O9.txt Data 1
Ca3ZrSi2O9.dia
(a)
I observed
I calculated
I difference
Background
Baghdadite
Intensity [counts]
Diffraction Angle [°2θ]

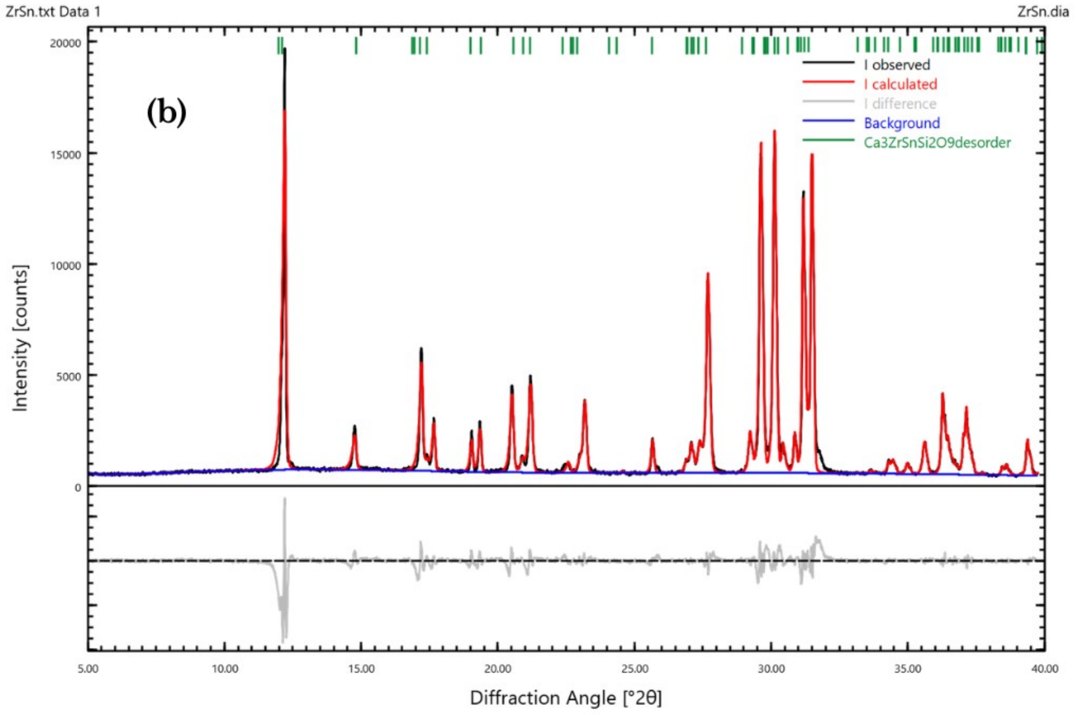
ZrSn.txt Data 1
ZrSn.dia
(b)
I observed
I calculated
I difference
Background
Ca3ZrSnSi2O9desorder
Intensity [counts]
Diffraction Angle [°2θ]

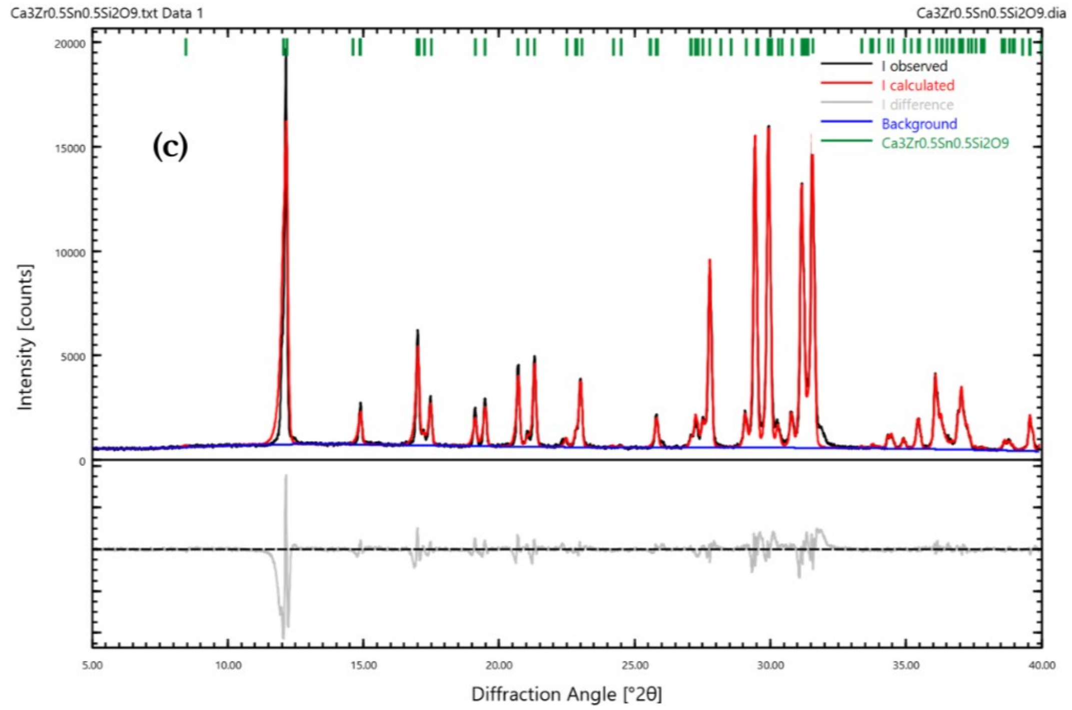
Ca3Zr0.5Sn0.5Si2O9.txt Data 1
Ca3Zr0.5Sn0.5Si2O9.dia
(c)
I observed
I calculated
I difference
Background
Ca3Zr0.5Sn0.5Si2O9
Intensity [counts]
Diffraction Angle [°2θ]

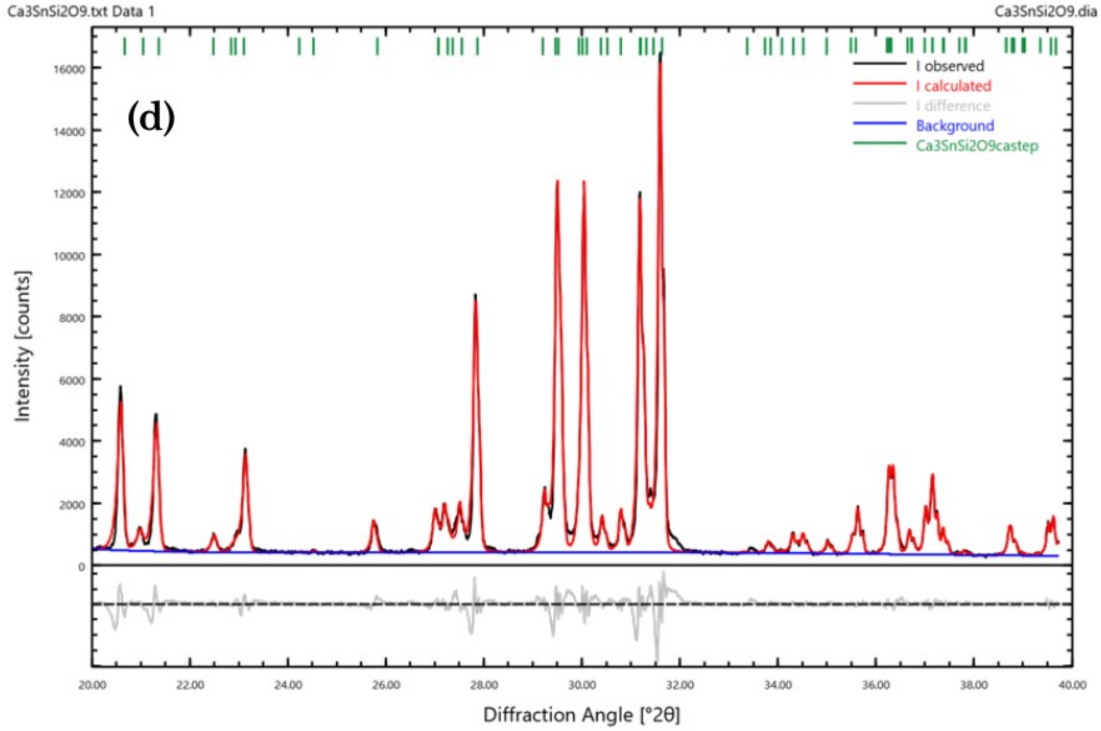
Ca3SnSi2O9.txt Data 1
Ca3SnSi2O9.dia
(d)
I observed
I calculated
I difference
Background
Ca3SnSi2O9castep
Intensity [counts]
Diffraction Angle [°2θ]

Figure S1. Rietveld refinement profilesof the XRD patterns for **(a)** $Ca_3ZrSi_2O_9$ **(b)** $Ca_3Zr_{0.5}Sn_{0.5}Si_2O_9$ refined using the average disordered $P2_1/c$ model **(c)** $Ca_3Zr_{0.5}Sn_{0.5}Si_2O_9$ refined using a representative ordered structural model and **(d)** $Ca_3SnSi_2O_9$ samples.

To better understand the structural description of $Ca_3Zr_{0.5}Sn_{0.5}Si_2O_9$, both disordered and ordered models were considered. The disordered model was used for the Rietveld refinement of the average crystal structure, whereas the ordered models were introduced to investigate possible local Sn arrangements and to provide suitable periodic models for first-principles calculations. In the disordered model, corresponding to the average monoclinic $P2_1/c$ structure, Zr and Sn share the same crystallographic site with equal occupancies. In the ordered models, two Sn atoms are explicitly distributed over the four octahedral sites, leading to three representative non-equivalent configurations denoted (1,2), (1,3), and (1,4) (Figure S2a–c).

The calculated XRD patterns shown in Figure S2(d) indicate that both the disordered model and the three ordered configurations reproduce the main experimental reflections, confirming that Sn substitution preserves the average monoclinic framework. However, additional weak reflections are observed near 8.5° for the ordered configurations. These reflections are more pronounced for the (1,3) and (1,4) arrangements, whereas their intensity is significantly reduced for the (1,2) configuration. Owing to its low intensity, this reflection may be masked by the experimental background and is therefore not clearly distinguishable in the experimental XRD pattern.

Among the investigated ordered models, the (1,2) configuration exhibits the closest agreement with the experimental diffraction data. Consequently, the (1,3) and (1,4) configurations were not considered further, while the representative (1,2) configuration was retained for subsequent analyses. In addition, Rietveld refinements based on both the average disordered $P2_1/c$ model

and the representative ordered (1,2) configuration yielded satisfactory agreement with the experimental diffraction pattern, supporting the suitability of these structural descriptions.

Since first-principles calculations of optical, thermoelectric, and photocatalytic properties require a periodic crystal model, the representative ordered (1,2) configuration was adopted for the subsequent calculations.

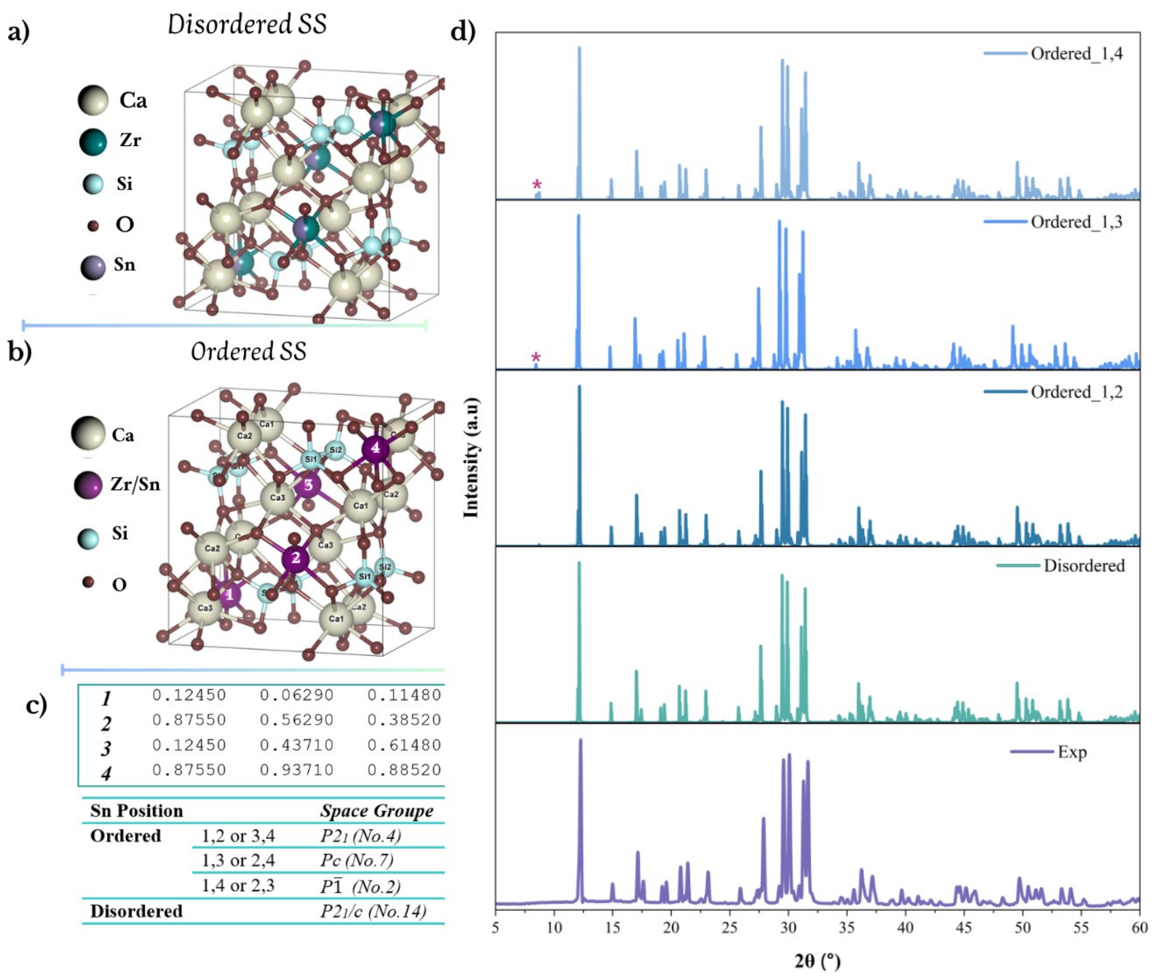

Figure S2. Ball and stick representation of **(a)** Disordered solid solution model, **(b)** Ordered solid solution model, **(c)** Fractional coordinates of the four octahedral sites (1–4) and the corresponding space groups, **(d)** Calculated XRD patterns (simulated from relaxed structures using VESTA) for the disordered model and the ordered (1,2), (1,3), and (1,4) configurations of $Ca_3Zr_{0.5}Sn_{0.5}Si_2O_9$, compared with experimental XRD patterns.

Table S1: Raman vibrational modes of $Ca_3Zr_{1-x}Sn_xSi_2O_9$ (x = 0, 0.50, 1), with comparison to literature data, mode assignments, and symmetry labels.

Raman $\tilde{\nu}$					Assignment	Symmetry of vibration
This work			Other work (x=0)			
x=0	x=0.5	x=1	Exp	Calc		
1029	1033	1034	1034	1032	a.s. Si−O(Si)	B_g
1007	1010	1014	1011	1010	s.s. Si−O(Si)	A_g
972	975	974	975	972	s.s. Si−O(Ca)	A_g
940,953	940,958	941,965	958,944	954,946	a.s. Si−O(Ca)	B_g,A_g
916	918	916	921	911	s.s. Si−O(Zr/Sn)	A_g
852	855	858	855	840	s.s. Si−O(Zr/Sn)	A_g
665	665	663	669	681	s.s. Zr−O(Zr)/ s.s. Sn−O(Sn)	B_g
620	620		624	652	b Zr−O−Zr	A_g
	603	603			b Sn−O−Sn	A_g
571	572	575	568	554	b Si−O−Si	B_g
538	543	546	542	531	b Si−O−Si	A_g
514	517		521	518	b Si−O−Zr	B_g
	485	484			b Si−O−Sn	B_g
	468	466			b Si−O−Sn	A_g
454	455		452	447	b Zr−O−Zr	A_g
430	432	430	433	429	b O−Si−O	A_g
403	403	406	409	411	b O−Si−O	A_g
371	370	373	376	383	b O−Zr−O/ b O−Sn−O	B_g
352	352	353	357	361	b O−Zr−O/ b O−Sn−O	A_g
318	319	325	321	314	r SiO_4	B_g
290	294	298	295	299	r SiO_4	A_g
259	251	265	262	258	b Si−O−Si	B_g
240	240	251	245	236	b Si−O−Zr/ b Si−O−Sn	B_g
210	211	211	214	214	b Si−O−Si	A_g
192	190	189	198	198	r SiO_4	A_g
	173	177			Si−O−Sn	A_g
168	169	166	171	178	s Ca−O	B_g
143	148	145	148	146	b Si−O−Si	B_g
123	124	121	122	124	Si−O−Zr/ Si−O−Sn	B_g
101	102	104	106	103	Si−O−Zr/ Si−O−Sn	A_g
94	95	95	97	94	Si−O−Zr/ Si−O−Sn	B_g

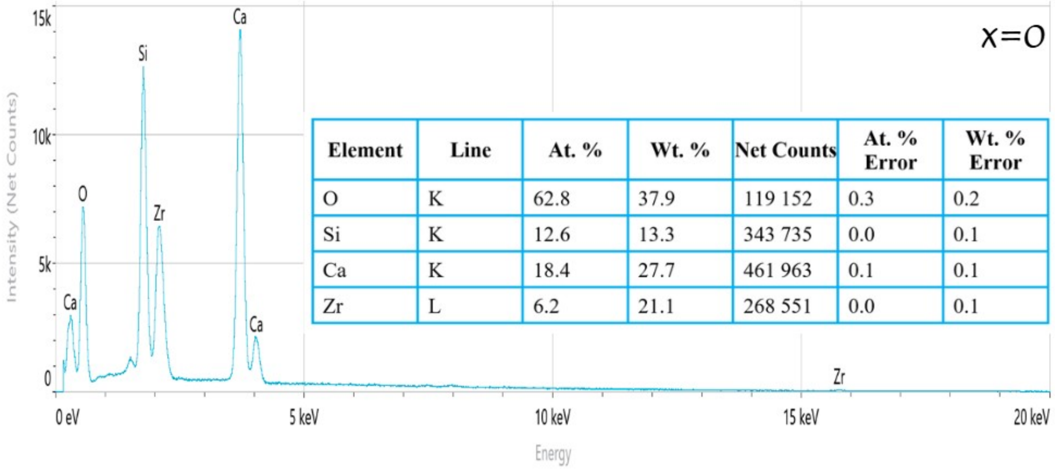

Element	Line	At. %	Wt. %	Net Counts	At. % Error	Wt. % Error
O	K	62.8	37.9	119 152	0.3	0.2
Si	K	12.6	13.3	343 735	0.0	0.1
Ca	K	18.4	27.7	461 963	0.1	0.1
Zr	L	6.2	21.1	268 551	0.0	0.1

	Atomic %		
**Element**	**Point 1**	**Point 2**	**Point 3**
O	66.5	66.3	63.0
Si	11.7	11.9	12.1
Ca	15.9	15.9	20.2
Zr	5.9	5.9	4.7

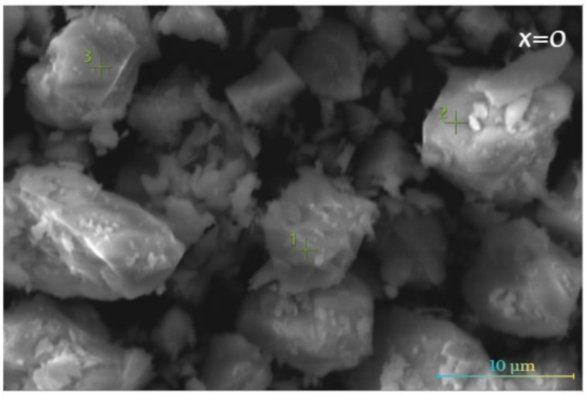

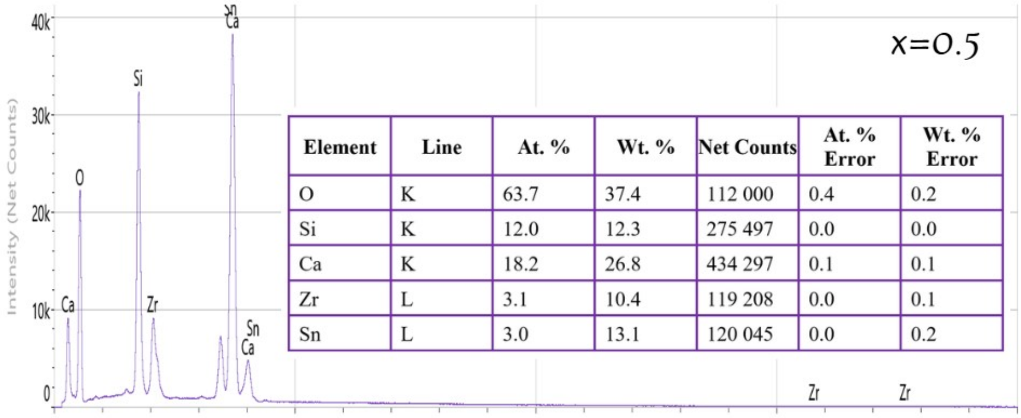

Element	Line	At. %	Wt. %	Net Counts	At. % Error	Wt. % Error
O	K	63.7	37.4	112 000	0.4	0.2
Si	K	12.0	12.3	275 497	0.0	0.0
Ca	K	18.2	26.8	434 297	0.1	0.1
Zr	L	3.1	10.4	119 208	0.0	0.1
Sn	L	3.0	13.1	120 045	0.0	0.2

	Atomic %		
**Element**	**Point 1**	**Point 2**	**Point 3**
O	74.5	45.7	66.2
Si	9.4	8.5	6.4
Ca	11.7	37.3	12.6
Zr	2.6	1.9	13.2
Sn	1.8	6.6	1.6

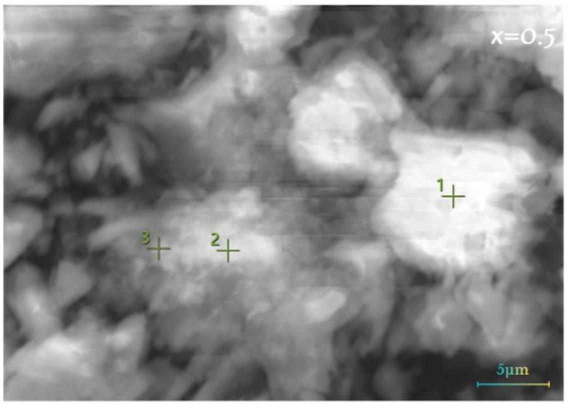

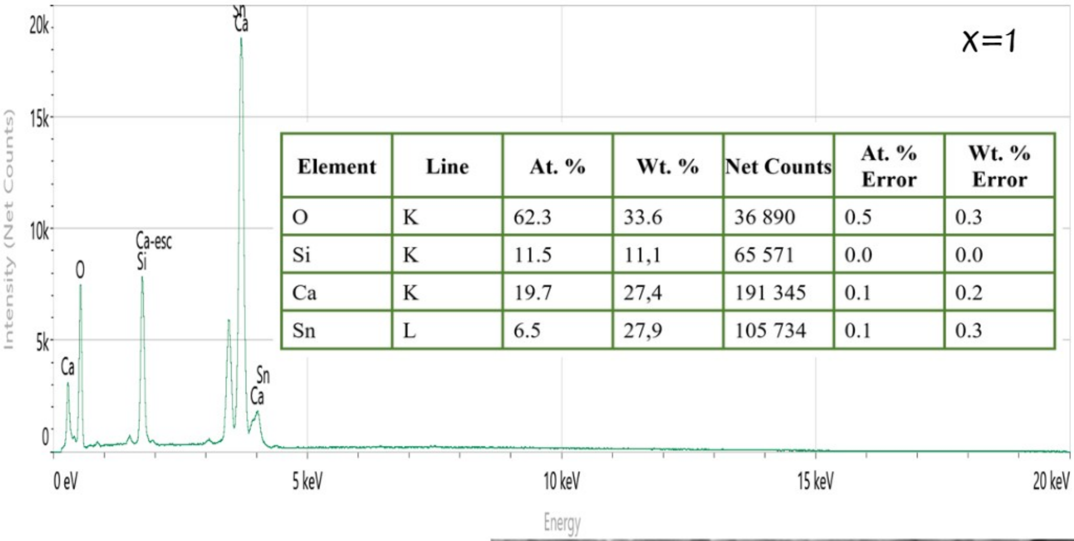

Element	Line	At. %	Wt. %	Net Counts	At. % Error	Wt. % Error
O	K	62.3	33.6	36 890	0.5	0.3
Si	K	11.5	11,1	65 571	0.0	0.0
Ca	K	19.7	27,4	191 345	0.1	0.2
Sn	L	6.5	27,9	105 734	0.1	0.3

	Atomic %		
**Element**	**Point 1**	**Point 2**	**Point 3**
O	62.5	62.3	66.8
Si	8.8	6.6	11.2
Ca	21.6	23.7	16.2
Sn	7.1	7.4	5.8

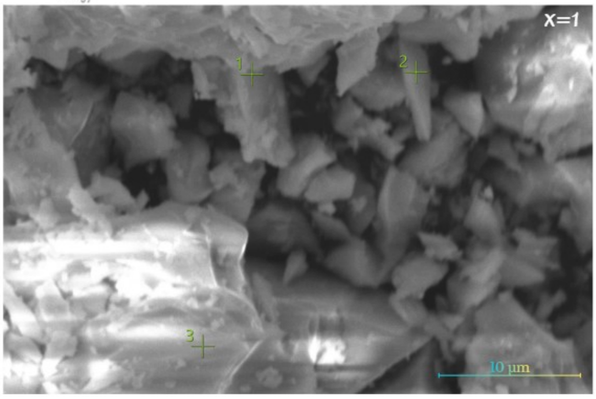

Figure S3. EDS analysis for $Ca_3Zr_{1-x}Sn_xSi_2O_9$ powders (x = 0, 0.5, 1).

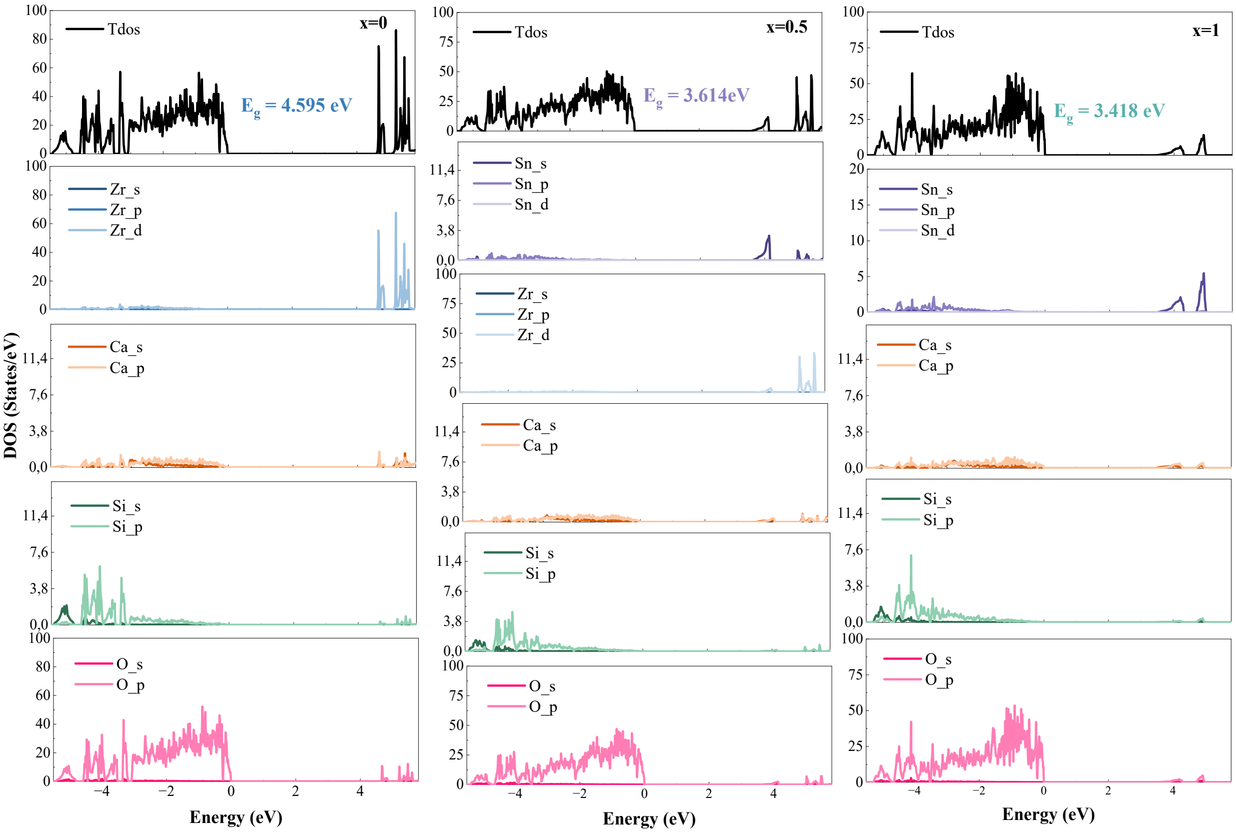

Figure S4. Calculated Total Density of States (TDOS) and Projected Density of States (PDOS) of $Ca_3Zr_{1-x}Sn_xSi_2O_9$ via GGA.
